# Evaluation of lung recruitment maneuvers in acute respiratory distress syndrome using computer simulation

**DOI:** 10.1186/s13054-014-0723-6

**Published:** 2015-01-12

**Authors:** Anup Das, Oana Cole, Marc Chikhani, Wenfei Wang, Tayyba Ali, Mainul Haque, Declan G Bates, Jonathan G Hardman

**Affiliations:** School of Engineering, University of Warwick, Library Road, Coventry, CV4 7AL UK; Anaesthesia & Critical Care Research Group, University of Nottingham, Derby Road, Nottingham, NG7 2UH UK

## Abstract

**Introduction:**

Direct comparison of the relative efficacy of different recruitment maneuvers (RMs) for patients with acute respiratory distress syndrome (ARDS) via clinical trials is difficult, due to the heterogeneity of patient populations and disease states, as well as a variety of practical issues. There is also significant uncertainty regarding the minimum values of positive end-expiratory pressure (PEEP) required to ensure maintenance of effective lung recruitment using RMs. We used patient-specific computational simulation to analyze how three different RMs act to improve physiological responses, and investigate how different levels of PEEP contribute to maintaining effective lung recruitment.

**Methods:**

We conducted experiments on five ‘virtual’ ARDS patients using a computational simulator that reproduces static and dynamic features of a multivariable clinical dataset on the responses of individual ARDS patients to a range of ventilator inputs. Three recruitment maneuvers (sustained inflation (SI), maximal recruitment strategy (MRS) followed by a titrated PEEP, and prolonged recruitment maneuver (PRM)) were implemented and evaluated for a range of different pressure settings.

**Results:**

All maneuvers demonstrated improvements in gas exchange, but the extent and duration of improvement varied significantly, as did the observed mechanism of operation. Maintaining adequate post-RM levels of PEEP was seen to be crucial in avoiding cliff-edge type re-collapse of alveolar units for all maneuvers. For all five patients, the MRS exhibited the most prolonged improvement in oxygenation, and we found that a PEEP setting of 35 cm H_2_O with a fixed driving pressure of 15 cm H_2_O (above PEEP) was sufficient to achieve 95% recruitment. Subsequently, we found that PEEP titrated to a value of 16 cm H_2_O was able to maintain 95% recruitment in all five patients.

**Conclusions:**

There appears to be significant scope for reducing the peak levels of PEEP originally specified in the MRS and hence to avoid exposing the lung to unnecessarily high pressures. More generally, our study highlights the huge potential of computer simulation to assist in evaluating the efficacy of different recruitment maneuvers, in understanding their modes of operation, in optimizing RMs for individual patients, and in supporting clinicians in the rational design of improved treatment strategies.

**Electronic supplementary material:**

The online version of this article (doi:10.1186/s13054-014-0723-6) contains supplementary material, which is available to authorized users.

## Introduction

Acute respiratory distress syndrome (ARDS) is a severe condition that affects around 1 in 10,000 people every year with life-threatening consequences [1]. The pathophysiology of ARDS is characterized by bronchoalveolar injury and alveolar collapse (atelectasis) [2-5]. The use of recruitment maneuvers (RMs) in ARDS to open up unstable, collapsed alveoli using a brief increase in transpulmonary pressure has become common practice in intensive care units [3], and a large variety of RMs has been proposed in the literature [3,6-12]. However, there remains a great deal of confusion regarding the optimal way to achieve and maintain alveolar recruitment in ARDS and, in many cases, the precise mode of action of particular RMs is not well understood [7,8,13,14].

The most frequently used recruitment maneuver in ARDS treatment is sustained inflation (SI) [8]. Studies have shown varying degrees of success, with several reporting post RM improvement in oxygenation [15,16] and reduction in lung atelectasis [17]. However, SI has also been shown to result in increased risk of hypotension [14,16] and barotrauma [18], decline in oxygenation [19] and has even been reported to be ineffective [20].

An alternative recruitment strategy that has been recently proposed is the prolonged recruitment maneuver (PRM) [21], in which positive end-expiratory pressure (PEEP) is fixed to a higher than baseline level and the positive inspiratory pressure is progressively increased. When PRM was compared with SI in an experimental model of mild acute lung injury (ALI) induced in a rat lung, it showed improved alveolar recruitment, gas exchange and a reduced level of lung damage. To date, however, no further evidence is available to support the use of PRM in ARDS patients.

The third RM considered in this paper is the maximal recruitment strategy (MRS), which was evaluated via patient trials in [6,22]. When this strategy was followed by ventilation with low tidal volumes and titrated PEEP, a median of 45% relative lung tissue recruitment was observed in quantitative computed tomography (CT) scan analysis. However, as noted in [13], the final PEEP levels applied at the end of the titration phase in the MRS resulted in inspiratory plateau pressures in the patient population of approximately 40 cm H_2_O, on average. This far exceeds the 28 cm H_2_O safety limit, which had been associated with increased inflammatory response in a previous study [23], and even the 30 cm H_2_O cutoff proposed by the ARDS Network. As noted in [6], it seems very likely that the MRS caused high degrees of alveolar stress and strain in some patients.

In this study, we employ a high-fidelity computational simulator that reproduces the static and dynamic characteristics of several ARDS patients, to (a) compare the efficacy of the three RMs described above in improving key patient parameters describing oxygenation, carbon dioxide (CO_2_) retention and dynamic compliance and (b) investigate the effects of different PEEP settings in maintaining effective lung recruitment across a representative ARDS patient spectrum. Our central hypothesis is that computational simulation can be used to evaluate and understand the mode of operation of RMs for ARDS patients.

## Methods

### The computational simulator

The simulation model considered in this study is an extended MATLAB™ implementation of several physiological models originally developed within the Nottingham Physiology Simulator [24-26]. The core models in the simulator have been designed to represent a dynamic *in vivo* cardio-vasculo-pulmonary state using a set of mass-conserving equations based on well-established physiological principles. The model simulates a lung divided into 100 alveolar compartments, with each compartment having a corresponding set of parameters accounting for stiffness, threshold opening pressures (TOPs) and extrinsic pressures as well as airway and vascular resistances. Recruitment is modeled as a time-dependent process [9,27] by the introduction of a ‘time-constant’ parameter (τ_c_) for each collapsed alveolar compartment, denoting the time it takes for the collapsed alveolus to open after a threshold pressure has been reached. The mathematical principles and equations on which the simulator is based have been detailed in previous studies [25,26], which have also validated the simulator’s ability to accurately represent pulmonary disease states. Full details are provided in an additional file (see Additional file 1).

### Computer simulation of RM protocols

**Figure 1:**
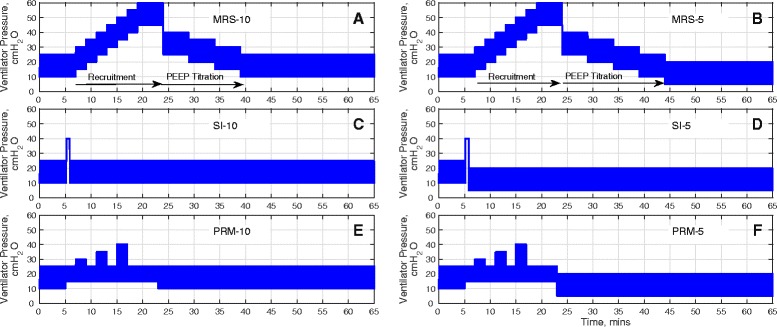
**Pressure waveform supplied by the mechanical ventilator over the simulation time for all the RMs implemented in this study. (A)** Maximal recruitment strategy with a final PEEP value of 10 cm H_2_O (MRS-10), **(B)** Maximal recruitment strategy with a final PEEP value of 5 cm H_2_O (MRS-5), **(C)** Sustained inflation with a final PEEP value of 10 cm H_2_O (SI-10), **(D)** Sustained inflation with a final PEEP value of 5 cm H_2_O (SI-5), **(E)** Prolonged recruitment maneuver with a final PEEP value of 10 cm H_2_O (PRM-10), **(F)** Prolonged recruitment maneuver with a final PEEP value of 5 cm H_2_O (PRM-5). From the plots, pressure at the end of expiration (that is the minimum pressure during a breath or in this case the PEEP setting) and peak inspiratory pressure (set by the ventilator) can be inferred. The MRS plots (A and B) indicate the recruitment and the PEEP titration phases. PEEP, positive end-expiratory pressure; RM, recruitment maneuver.

Each protocol consists of a pre-RM stage, the RM stage and a post-RM stage. During the pre-RM stage, the *in silico* patients were subjected to identical end-expiratory pressures (10 cm H_2_O) and identical inspiratory pressure (15 cm H_2_O above PEEP). At the post RM stage, the inspiratory pressure is maintained at 15 cm H_2_O above PEEP while the PEEP is set to either 5 cm H_2_O or 10 cm H_2_O. Throughout the protocols, the fraction of inspired oxygen (F_I_O_2_), the hemoglobin level (Hb) and the cardiac output (CO) was maintained at the value suggested by the source data (which were used to configure each virtual patient), while the inspiratory to expiratory (IE) ratio and ventilation rate were chosen based on the available patient data (see below). Three RMs from the published literature were implemented in the simulator as detailed below and illustrated in Figure 1.

#### Maximal recruitment strategy (MRS)

The MRS [6] consists of two-minute steps of tidal ventilation in pressure-controlled mode, with a fixed driving pressure of 15 cm H_2_O (above PEEP). During the recruitment phase, PEEP was increased from 5 cm H_2_O to a maximum of 45 cm H_2_O in steps of 5 cm H_2_O, with each step lasting 2 minutes. During the PEEP titration phase, the PEEP is set to 25 cm H_2_O and then further reduced by 5 cm H_2_O in steps to the end-maneuver PEEP, with each step lasting 5 minutes. The end-maneuver PEEP was set to 5 cm H_2_O (MRS-5) or 10 cm H_2_O (MRS-10).

#### Sustained inflation (SI)

This SI [28] was simulated as a sustained pulmonary inflation maneuver, with a positive ventilator pressure of 40 cm H_2_O applied for 40 seconds. The end-maneuver PEEP was set to 5 cm H_2_O (SI-5) or 10 cm H_2_O (SI-10).

#### Prolonged recruitment maneuver (PRM)

The inspiratory pressure in the PRM [21] was progressively increased every 2 minutes in steps of 5 cm H_2_O from 15 cm H_2_O to 25 cm H_2_O, above a fixed PEEP of 15 cm H_2_O. The end-maneuver PEEP was set to 5 cm H_2_O (PRM-5) or 10 cm H_2_O (PRM-10).

The effectiveness of each RM was assessed via a common set of clinically relevant indicators: improvement in oxygenation (represented through the ratio of partial pressure of oxygen in arterial blood to the fraction of oxygen in inspired air, (PaO_2_/F_I_O_2_)), the change in peak alveolar pressures (P_peak_, representing the risk of barotrauma, calculated as the average of the maximum pressure in the most highly pressurized 20% of the 100 alveolar compartments, over the entire maneuver) the dynamic compliance, and the change in arterial carbon dioxide partial pressure (PaCO_2_).

### Configuring the simulator to static and dynamic ARDS patient data

The model was configured to fit data from individual ARDS patients in two stages. In the first stage, the model was matched to static data reported by Nirmalan and colleagues [29], which listed arterial and mixed venous blood gas values and cardiac output measurements taken from patients treated for ARDS. The data obtained from Nirmalan [29] contain only static measurements and thus do not provide information concerning dynamic processes such as recruitment. Therefore, a second matching was done to determine the value of τ_c_ (representing the time it could take for collapsed alveoli to open after a threshold pressure is reached, (see Additional file 1)) for each compartment so as to best fit the data provided by Chiumello and colleagues [30], which reports blood gas measurements in ARDS patients over a 60-minute period as a result of step changes in PEEP. In both stages, advanced optimization algorithms were used to find physiologically realistic values of model parameters that best fit the available data – full details are provided in an additional file (see Additional file 2).

## Results

### Reproduction of ARDS patient data

**Figure 2:**
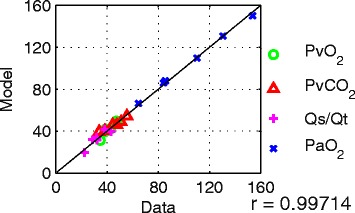
**Model outputs versus data recorded in [** 29 **] for five patients (Table** 1 **).** R, Pearson’s correlation coefficient. Units for PvO_2,_ PvCO_2_ and PaO_2_ are in mmHg. PaO_2_, partial pressure of oxygen in arterial blood; PvCO_2_, partial pressure of carbon dioxide in venous blood; PvO_2_, partial pressure of oxygen in venous blood; Qs/Qt, shunt fraction.

**Figure 3:**
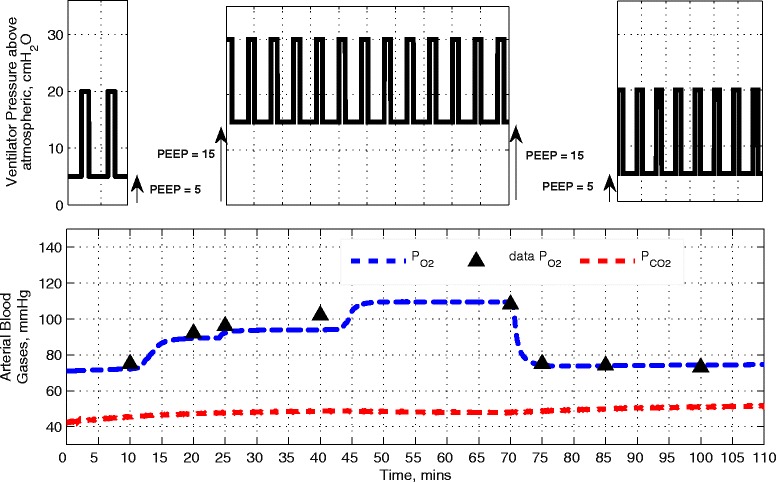
**Model PaO**_**2**_ **fitted to data obtained from [** 36 **], resulting from changes in PEEP from 5 to 15 cm H**_**2**_ **O and then back to 5 cm H**_**2**_ **O.** PaO_2_, partial pressure of oxygen in arterial blood; PEEP, positive end-expiratory pressure.

The results of matching the model to static data from five patients from [29] are given in Table 1. The combined results for the five patients show an excellent linear correlation (Figure 2, with *r* = 0.997 (*P* <0.0001)) between the model-generated outputs and observations in the data. As shown in Figure 3, the outputs of the simulator also provide an extremely close fit to the dynamic data reported in [30].

**Table 1:** **Results of model fitting for five ARDS patients**

		**Patient A**		**Patient B**		**Patient C**		**Patient D**		**Patient E**	
Parameters obtained from data	Hb (g dl^−1^)	10.5		10.8		11.5		9.8		9	
	CO (l min^−1^)	11.1		7.2		5.6		7.7		5.9	
	F_I_O_2_	0.8		0.9		0.5		0.8		1	
Parameters obtained through optimization	VR (b min^−1^)	12.25		12.14		16.04		17.68		17.0	
	IE	0.28		0.25		0.38		0.38		0.43	
	RQ	0.6		0.6		0.9		0.7		0.61	
	VO_2_ (ml min^−1^)	294.3		300		200		257.2		246.8	
	% of compartments collapsed	26		29		12		20		21	
Parameters fixed for RM trials	P_v_ (cm H_2_O)	15		15		15		15		15	
	PEEP (cm H_2_O)	5		5		5		5		5	
		Data	Model	Data	Model	Data	Model	Data	Model	Data	Model
Results of fitting the model to the data	PvO_2_ (mmHg)	47.3	49.5	38.3	39.4	48	45.4	42.83	42.2	34.5	30.3
	PvCO_2_ (mmHg)	44.4	46.2	55.5	54.4	47.6	49.9	51	48.8	33.82	36.07
	Qs/Qt (%)	28.6	31.8	31.7	33.9	22.6	19.4	32.3	31.4	43.1	39.14
	PaO_2_ (mmHg)	153.7	149.9	85.5	87.9	130.5	129.6	110.3	109.6	64.5	64.95

ARDS, acute respiratory distress syndrome; Hb, hemoglobin; CO, cardiac output; F_I_O_2_, fraction of inspired oxygen; VR, ventilation rate; IE, inspiratory to expiratory ratio; RQ, respiratory quotient; VO_2_, oxygen consumption; Pv, ventilator pressure; PEEP, positive end-expiratory pressure; PvO_2_, partial pressure of oxygen in venous blood; PvCO_2_, partial pressure of carbon dioxide in venous blood; Qs/Qt, shunt fraction; PaO_2_, partial pressure of oxygen in arterial blood.

### Comparative evaluation of RMs for a moderate ARDS state

**Figure 4:**
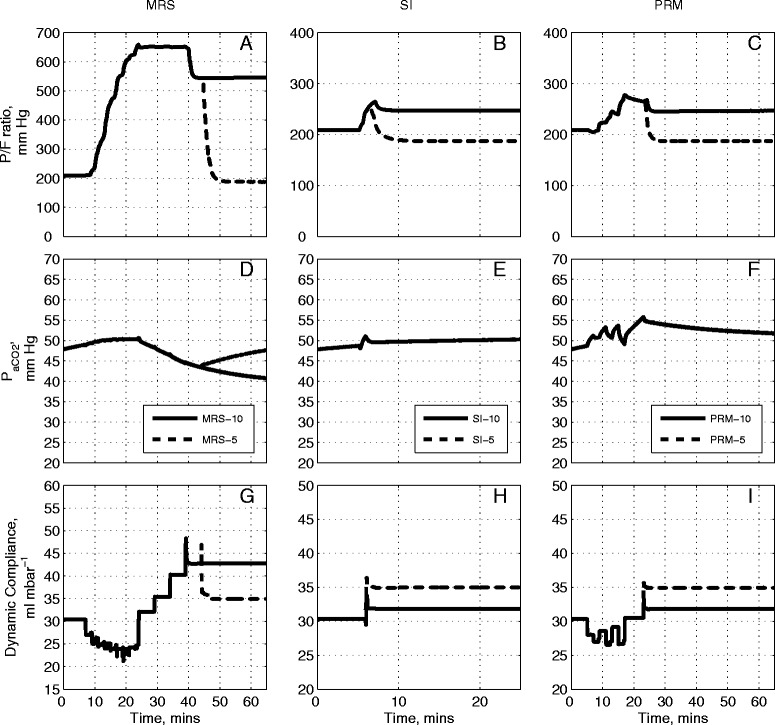
**Model outputs for Patient A under the recruitment maneuvers given in Figure** 1 **.** The continuous lines are outputs for PEEP = 10 cm H_2_O and dashed lines are outputs for PEEP = 5 cm H_2_O. The plot has nine panels: **(A)** the partial pressure of O_2_ to fraction of inhaled O_2_ ratio (mmHg) under MRS-10 and MRS-5, **(B)** the partial pressure of O_2_ to fraction of inhaled O_2_ ratio (mmHg) under SI-10 and SI-5, **(C)** the partial pressure of O_2_ to fraction of inhaled O_2_ ratio (mmHg) under PRM-10 and PRM-5, (**D)** the partial pressure of CO_2_ in arterial blood (mmHg) under MRS-10 and MRS-5, **(E)** the partial pressure of CO_2_ in arterial blood (mmHg) under SI-10 and SI-5, **(F)** the partial pressure of CO_2_ in arterial blood (mmHg) under PRM-10 and PRM-5, **(G)** dynamic compliance of the lung (ml mbar^−1^) under MRS-10 and MRS-5, **(H)** dynamic compliance of the lung (ml mbar^−1^) under SI-10 and SI-5, **(I)** dynamic compliance of the lung (ml mbar^−1^) under PRM-10 and PRM-5. CO_2_, carbon dioxide; MRS, maximum recruitment strategy; O_2_, oxygen; PEEP, positive end-expiratory pressure; PRM, prolonged recruitment maneuver; SI, sustained inflation.

Figure 4 presents results obtained by applying the three RMs to the model configured to match Patient A in Table 1. According to the Berlin definition [31], patient A was classified as suffering from moderate ARDS (PaO_2_/F_I_O_2_ of 192.13 mmHg, see Table 1). As seen in Figure 4A, the application of the MRS causes the PaO_2_/F_I_O_2_ to increase by more than 400 mmHg. It remains raised throughout the recruitment stage and plateaus at its maximal value of 650 mmHg, continuing through to the PEEP titration phase. PaO_2_/F_I_O_2_ started to fall at the 40th minute interval, corresponding to the PEEP titration step to 10 cm H_2_O. Under MRS-5, the reduction of PEEP to 5 cm H_2_O resulted in a further fall of PaO_2_/F_I_O_2_ to near pre-RM levels of 200 mmHg. The SI maneuver resulted in a modest increase in the PaO_2_/F_I_O_2_ ratio (Figure 4B) from 200 mmHg to approximately 260 mmHg. A final PEEP value of 10 cm H_2_O was sufficient to maintain PaO_2_/F_I_O_2_ at 250 mmHg, while reducing the final PEEP value to 5 cm H_2_O produced a final PaO_2_/F_I_O_2_ of only 190 mmHg. Results of the application of the PRM (Figure 4C) show the PaO_2_/F_I_O_2_ ratio rising from 200 mmHg to a maximum value of 270 mmHg at the highest inspiratory pressure at the 15th minute interval. For PRM-10, the final PaO_2_/F_I_O_2_ remained at 250 mmHg whereas the PaO_2_/F_I_O_2_ dropped to below 200 mmHg for PRM-5. In all the RM protocols, a post-RM stage PEEP of 10 cm H_2_O results in a significantly higher PaO_2_/F_I_O_2_ than that obtained with the pre-RM stage PEEP of 10 cm H_2_O.

Changes in PaCO_2_ are useful indicators of the pathological state of the lung, revealing the effectiveness of gas exchange, the presence of dead space, and acid-base balance of the blood. In Figure 4D, as the MRS begins, PaCO_2_ rises slightly, from 47 mmHg to 50 mmHg (until the 15th minute interval) where PaCO_2_ settles over the following 10 minutes (corresponding to the MRS reaching the peak ventilator pressure; see Figure 1A and B). This was followed by a reduction in PaCO_2_ until the 45th minute interval (as the PEEP titration stage ends) at which point the PaCO_2_ value achieved by MRS-5 deviates from that of MRS-10. The MRS-10 continues to make PaCO_2_ fall further whereas PaCO_2_ under MRS-5 returns to the level observed at the pre-RM stage. With SI, a slight rise in PaCO_2_ can be seen but the overall variation is minimal (Figure 4E) for both SI maneuvers (SI-5 and SI-10). Under the PRM (Figure 4F), from the baseline PaCO_2_ value of 47 mmHg, the PaCO_2_ rises and fluctuates around 52 mmHg until the 18th minute interval at which point it rises to a maximum value at 55 mmHg. The rest of the maneuver produced a slight drop in PaCO_2_ to a final value of 52 mmHg. No difference was observed between the PaCO_2_ values produced by PRM-10 and PRM-5 over the entire maneuver.

The MRS produced notable changes in the dynamic compliance of the lung (see Figure 4G). Beginning from an initial value of 30 ml mbar^−1^, as PEEP is increased, compliance fell to a minimum of 21 ml mbar^−1^. During PEEP titration, compliance gradually increased again to a peak of 48 ml mbar^−1^, the maximal value coinciding with the last step of the PEEP reduction. At this point, the higher final PEEP setting of MRS-10 resulted in the final dynamic compliance value settling at 43 ml mbar^−1^ while MRS-5 resulted in a lower final dynamic compliance value of 35 ml mbar^−1^. The SI maneuver was accompanied by a sharp increase in compliance; with SI-10 settling at a value of 32 ml mbar^−1^ and SI-5 settling at a slightly higher value of 35 ml mbar^−1^, as shown in Figure 4H. Finally, the PRM resulted in an overall drop in lung compliance while the maneuver is under progress, followed by an increase back to the baseline value upon cessation of the maneuver (Figure 4I). Under PRM-10, the final dynamic compliance value was recorded at 32 ml mbar^−1^ while under SI-5, the dynamic compliance settled at a higher value of 35 ml mbar^−1^.

### Comparative evaluation of three RMs for a severe ARDS state

**Figure 5:**
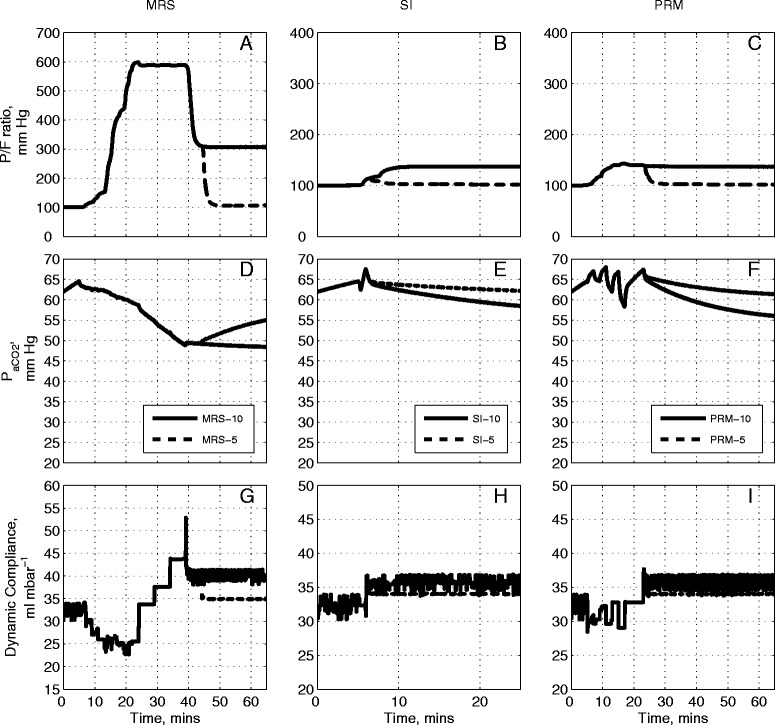
**Model outputs for Patient B under the recruitment maneuvers given in Figure** 1 **.** The continuous lines are outputs for PEEP = 10 cm H_2_O and dashed lines are outputs for PEEP = 5 cm H_2_O. The plot has nine panels: **(A)** the partial pressure of O_2_ to fraction of inhaled O_2_ ratio (mmHg) under MRS-10 and MRS-5, **(B)** the partial pressure of O_2_ to fraction of inhaled O_2_ ratio (mmHg) under SI-10 and SI-5, **(C)** the partial pressure of O_2_ to fraction of inhaled O_2_ ratio (mmHg) under PRM-10 and PRM-5, **(D)** the partial pressure of CO_2_ in arterial blood (mmHg) under MRS-10 and MRS-5, **(E)** the partial pressure of CO_2_ in arterial blood (mmHg) under SI-10 and SI-5, **(F)** the partial pressure of CO_2_ in arterial blood (mmHg) under PRM-10 and PRM-5, **(G)** dynamic compliance of the lung (ml mbar^−1^) under MRS-10 and MRS-5, **(H)** dynamic compliance of the lung (ml mbar^−1^) under SI-10 and SI-5, **(I)** dynamic compliance of the lung (ml mbar^−1^) under PRM-10 and PRM-5. CO_2_, carbon dioxide; MRS, maximum recruitment strategy; O_2_, oxygen; PEEP, positive end-expiratory pressure; PRM, prolonged recruitment maneuver; SI, sustained inflation.

Figure 5 shows the results of the application of the RMs to Patient B (from Table 1), who was classified under the Berlin definition [31] as suffering from severe ARDS (baseline PaO_2_/F_I_O_2_ was less than 100 mmHg, see Table 1). The initial PaCO_2_ of 62 mmHg was also considerably higher than that of Patient A (47 mmHg), indicating severe hypercapnia. The increased severity of the initial ARDS state leads to reduced improvement in outcomes in each case; however, the relative efficacy of the different RMs was similar to that observed with Patient A. Also, as in the case of Patient A, reduction of the final PEEP value from 10 cm H_2_O to 5 cm H_2_O resulted in all improvements in recruitment being lost on completion of each maneuver.

### Comparative evaluation on three other patients

**Figure 6:**
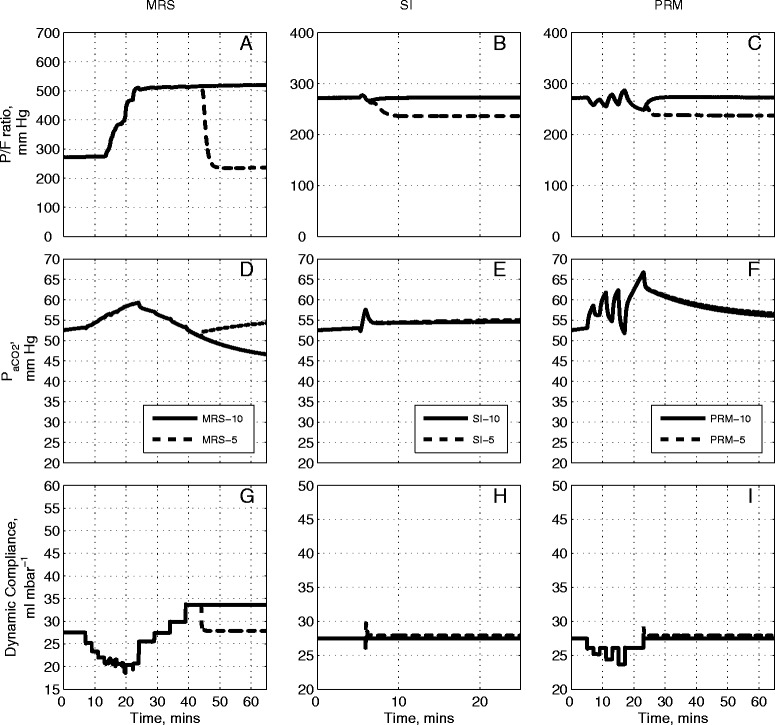
**Model outputs for Patient C under the recruitment maneuvers given in Figure** 1 **.** The continuous lines are outputs for PEEP = 10 cm H_2_O and dashed lines are outputs for PEEP = 5 cm H_2_O. The plot has nine panels: **(A)** the partial pressure of O_2_ to fraction of inhaled O_2_ ratio (mmHg) under MRS-10 and MRS-5, **(B)** the partial pressure of O_2_ to fraction of inhaled O_2_ ratio (mmHg) under SI-10 and SI-5, **(C)** the partial pressure of O_2_ to fraction of inhaled O_2_ ratio (mmHg) under PRM-10 and PRM-5, **(D)** the partial pressure of CO_2_ in arterial blood (mmHg) under MRS-10 and MRS-5, **(E)** the partial pressure of CO_2_ in arterial blood (mmHg) under SI-10 and SI-5, **(F)** the partial pressure of CO_2_ in arterial blood (mmHg) under PRM-10 and PRM-5, **(G)** dynamic compliance of the lung (ml mbar^−1^) under MRS-10 and MRS-5, **(H)** dynamic compliance of the lung (ml mbar^−1^) under SI-10 and SI-5, **(I)** dynamic compliance of the lung (ml mbar^−1^) under PRM-10 and PRM-5. CO_2_, carbon dioxide; MRS, maximum recruitment strategy; O_2_, oxygen; PEEP, positive end-expiratory pressure; PRM, prolonged recruitment maneuver; SI, sustained inflation.

**Figure 7:**
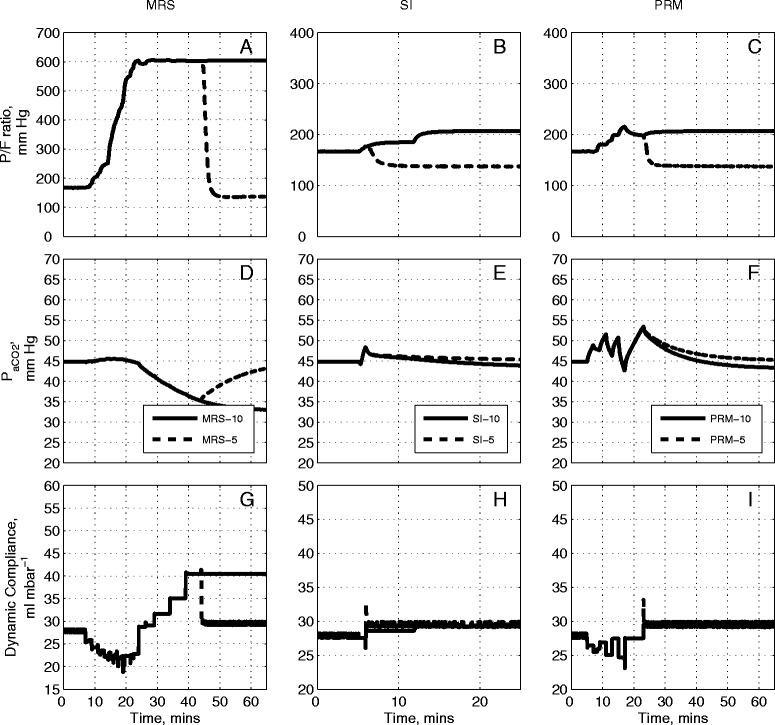
**Model outputs for Patient D under the recruitment maneuvers given in Figure** 1 **.** The continuous lines are outputs for PEEP = 10 cm H_2_O and dashed lines are outputs for PEEP = 5 cm H_2_O. The plot has nine panels: **(A)** the partial pressure of O_2_ to fraction of inhaled O_2_ ratio (mmHg) under MRS-10 and MRS-5, **(B)** the partial pressure of O_2_ to fraction of inhaled O_2_ ratio (mmHg) under SI-10 and SI-5, **(C)** the partial pressure of O_2_ to fraction of inhaled O_2_ ratio (mmHg) under PRM-10 and PRM-5, **(D)** the partial pressure of CO_2_ in arterial blood (mmHg) under MRS-10 and MRS-5, **(E)** the partial pressure of CO_2_ in arterial blood (mmHg) under SI-10 and SI-5, **(F)** the partial pressure of CO_2_ in arterial blood (mmHg) under PRM-10 and PRM-5, **(G)** dynamic compliance of the lung (ml mbar^−1^) under MRS-10 and MRS-5, **(H)** dynamic compliance of the lung (ml mbar^−1^) under SI-10 and SI-5, **(I)** dynamic compliance of the lung (ml mbar^−1^) under PRM-10 and PRM-5. CO_2_, carbon dioxide; MRS, maximum recruitment strategy; O_2_, oxygen; PEEP, positive end-expiratory pressure; PRM, prolonged recruitment maneuver; SI, sustained inflation.

**Figure 8:**
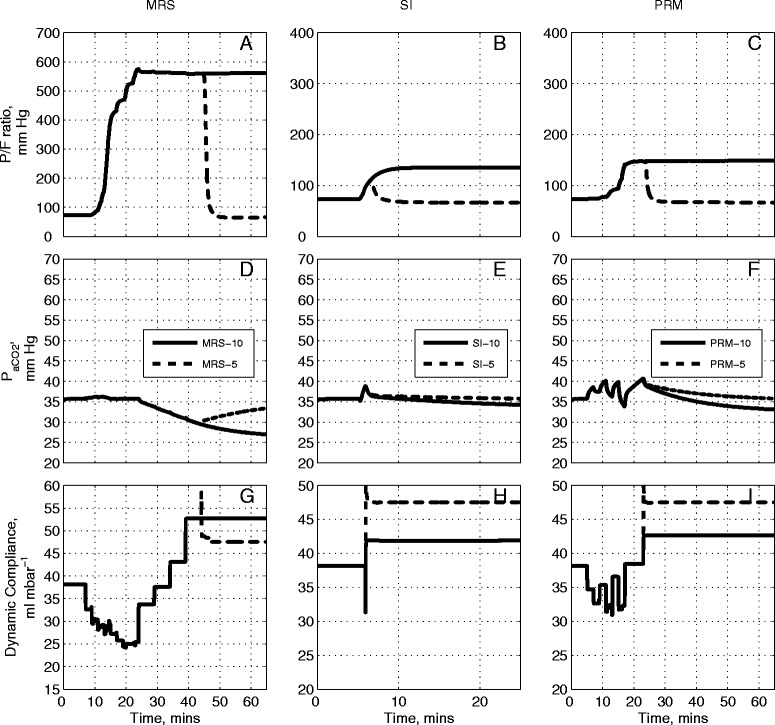
**Model outputs for Patient E under the recruitment maneuvers given in Figure** 1 **.** The continuous lines are outputs for PEEP = 10 cm H_2_O and dashed lines are outputs for PEEP = 5 cm H_2_O. The plot has nine panels: **(A)** the partial pressure of O_2_ to fraction of inhaled O_2_ ratio (mmHg) under MRS-10 and MRS-5, **(B)** the partial pressure of O_2_ to fraction of inhaled O_2_ ratio (mmHg) under SI-10 and SI-5, **(C)** the partial pressure of O_2_ to fraction of inhaled O_2_ ratio (mmHg) under PRM-10 and PRM-5, **(D)** the partial pressure of CO_2_ in arterial blood (mmHg) under MRS-10 and MRS-5, **(E)** the partial pressure of CO_2_ in arterial blood (mmHg) under SI-10 and SI-5, **(F)** the partial pressure of CO_2_ in arterial blood (mmHg) under PRM-10 and PRM-5, **(G)** dynamic compliance of the lung (ml mbar^−1^) under MRS-10 and MRS-5, **(H)** dynamic compliance of the lung (ml mbar^−1^) under SI-10 and SI-5, **(I)** dynamic compliance of the lung (ml mbar^−1^) under PRM-10 and PRM-5. CO_2_, carbon dioxide; MRS, maximum recruitment strategy; O_2_, oxygen; PEEP, positive end-expiratory pressure; PRM, prolonged recruitment maneuver; SI, sustained inflation.

From Table 1, and using the Berlin definition, [31], Patient C would be considered as suffering from mild ARDS (baseline PaO_2_/F_I_O_2_ was equal 261 mmHg), Patient D as suffering from moderate ARDS (baseline PaO_2_/F_I_O_2_ was equal to 138 mmHg) and Patient E suffering from severe ARDS (baseline PaO_2_/F_I_O_2_ was equal to 65 mmHg). For patients C, D and E (Figures 6, 7 and 8 respectively) the MRS followed the pattern of the outcomes generated in Patients A and B. Unlike with Patients A and B however, the post-RM values of PaO_2_/F_I_O_2_ were maintained at their maximum values for all three patients for a final PEEP setting of 10 cm H_2_O in MRS-10 (Figures 6A, 7A, 8A). The SI maneuver produced a modest change in overall PaCO_2_ (Figures 6E, 7E, 8E) for all three patients.

In Patient C (Figures 6B and C) for a post-RM PEEP setting of 10 cm H_2_O (SI-10 and PRM-10), no improvement was observed between pre-RM and post-RM values of PaO_2_/F_I_O_2_ (Figure 6B and C). With the post-RM PEEP of 5 cm H_2_O (SI-5 and PRM-5), the PaO_2_/F_I_O_2_ was reduced from its pre-RM value of 270 mmHg to a post-RM value of 240 mmHg. There was little noticeable change in initial and final values for the dynamic compliance during the SI and PRM maneuvers (Figures 6H and I).

### Comparing the risk of lung injury

A major issue with all RMs is the high ventilator pressures delivered to the patient, which can contribute to ventilator-associated lung injury (VALI). Table 2 shows the peak alveolar pressure (P_peak,_ calculated as the average of the maximum pressure in the most highly pressurised 20% of the 100 alveolar compartments over the entire maneuver) that were delivered to each patient during the three recruitment maneuvers with final PEEP values of 10 cm H_2_O. It is evident from Table 2 that although the MRS results in higher values of P_peak_ than those produced by the other RMs, the resulting improvement in PaO_2_/F_I_O_2_ is considerably better than that achieved by the other two maneuvers in all five patients.

**Table 2:** **Results of airway pressures**

	**RM**	Δ**PO**_**2**_ **, cm H**_**2**_ **O**	**P**_**peak**_ **, cm H**_**2**_ **O**
**Patient A**	MRS-10	513.21	57.09
	SI-10	84.10	40.01
	PRM-10	98.10	32.75
**Patient B**			
	MRS-10	613.94	58.04
	SI-10	49.55	40.03
	PRM-10	56.66	35.18
**Patient C**			
	MRS-10	207.59	59.40
	SI-10	31.65	40.04
	PRM-10	38.71	38.35
**Patient D**			
	MRS-10	510.70	57.93
	SI-10	75.88	40.01
	PRM-10	85.21	35.05
**Patient E**			
	MRS-10	694.58	57.09
	SI-10	95.27	40.01
	PRM-10	113.42	32.75

RM, recruitment maneuver; ΔPO_2_, difference between maximum PaO_2_ and baseline PaO_2_; P_peak_, peak alveolar pressure; MRS-10, maximal recruitment strategy with a final PEEP value of 10 cm H_2_O; SI-10, sustained inflation with a final PEEP value of 10 cm H_2_O; PRM-10, prolonged recruitment maneuver with a final PEEP value of 10 cm H_2_O; PaO_2_, partial pressure of arterial oxygen; PEEP, positive end-expiratory pressure.

### Computing minimum necessary PEEP values for the MRS

**Figure 9:**
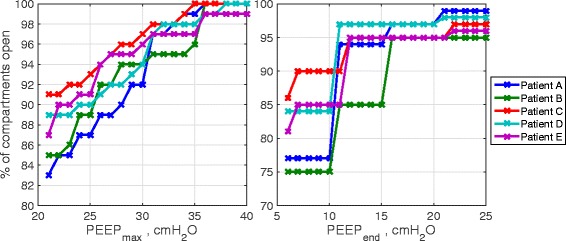
**The number of recruited compartments in each of the five patients with the maximum recruitment strategy for different values of (A) PEEP**_**max**_ **– the maximum value of PEEP at the end of the recruitment stage and (B) PEEP**_**end**_ **– the final value of PEEP at the end of titration stage.** PEEP, positive end-expiratory pressure.

**Figure 10:**
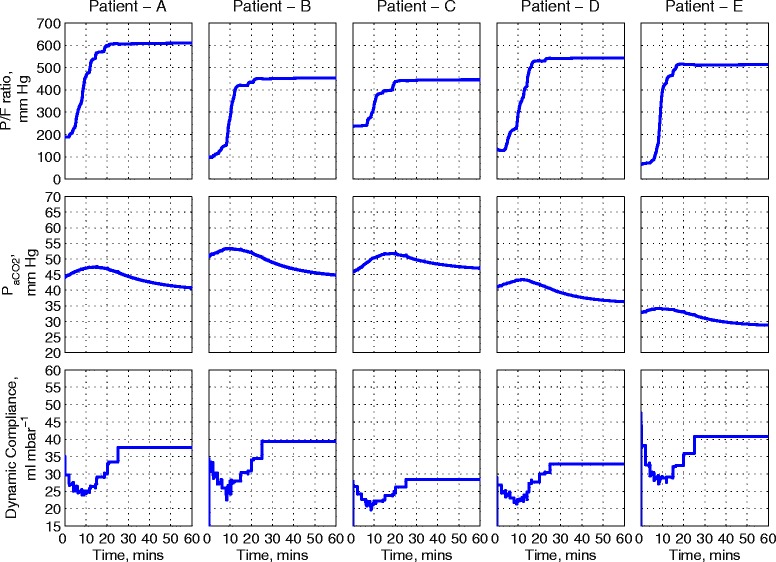
**The PaO**_**2**_ **/F**_**I**_ **O**_**2**_ **ratio, PaCO**_**2**_ **, and dynamic compliance plots of the five patients for the MRS implemented with a PEEP**_**max**_ **value of 31 cm H**_**2**_ **O and a PEEP**_**end**_ **value of 16 cm H**_**2**_ **O.** MRS, maximum recruitment strategy; PaCO_2_, partial pressure of arterial carbon dioxide; PaO_2_/F_I_O_2_, ratio of partial pressure of oxygen in arterial blood to fraction of oxygen in inspired air; PEEP, positive end-expiratory pressure; PEEP_end_, PEEP value at the end of simulation; PEEP_max_, maximum PEEP during a recruitment maneuver.

We next investigated whether the peak and final PEEP values originally proposed for the MRS in [6,22] are indeed the minimum values necessary to maintain effective lung recruitment in our virtual patients. It should be noted that a maximum PEEP of 45 cm H_2_O and a final PEEP of 25 cm H_2_O as reported in [6,22] were only recommended values and the authors proposed the use of CT scans to guide the selection of PEEP values. Figure 9A shows the percentage of alveolar compartments recruited by the MRS in each of the five patients for different peak values of PEEP (PEEP_max_). Figure 9B shows the percentage of alveolar compartments that remained open after completion of the MRS for different final values of PEEP (PEEP_end_). As shown in Figure 9A, a value of PEEP_max_ of 35 cm H_2_O with a fixed driving pressure of 15 cm H_2_O (above PEEP) was sufficient to achieve recruitment in 95% of alveolar compartments for all five patients. This value is significantly lower than the maximum PEEP value of 45 cm H_2_O suggested in the original publications proposing the MRS [6,22]. For PEEP_end_, a value of 16 cm H_2_O was required to maintain 95% recruitment in all five patients – this is also significantly lower than the maximum value of 25 cm H_2_O specified in [22], although it is slightly higher than the value of 10 cm H_2_O suggested in [6]. Implementation of the MRS with the minimum necessary peak and final PEEP values suggested by our analysis produced the results shown in Figure 10.

## Discussion

The marked improvement in PaO_2_/F_I_O_2_ seen in all five patients following the application of the MRS is striking, and might be explained as follows. During inspiration, a normal lung increases its volume uniformly, as almost all of its compartments have the same dynamic elastance and homogenous structure. This is not true in the case of diseased lungs, however. Gattinoni and colleagues [32] have shown that the ARDS lung is characterized by a small functional volume termed the ‘baby lung’, which stays open throughout the respiratory cycle. At end-expiration, an adequate PEEP can maintain some of the recruited lung regions open for the next respiratory cycle to take place. Therefore, selecting an appropriate recruitment strategy and modifying the PEEP applied at the end of an intensive ventilation period should increase and maintain the size of the baby lung and contribute to improving the gas exchange. As shown in Figure 9, in the simulator almost 100% of the alveolar compartments are recruited during a maximal recruitment maneuver, while 80% of the lung is recruited during a PRM or a SI maneuver. However, the last two maneuvers are followed by an immediate derecruitment, whereas the MRS is able to maintain alveolar recruitment for a significant period of time.

It should be noted that for each patient, apart from the variation in the ventilator pressure during the RM, no other parameters such as the ventilator settings of IE ratio, ventilation rate (VR), or F_I_O_2_ were changed. This explains the difference in outputs (Figure 9) between patients who each had most of their alveolar units recruited (Figure 8).

The results also display the presence of several interesting phenomena within the lung during the implementation of the various RM. For all RMs, a PEEP level of 10 cm H_2_O with identical driving pressure at the end of recruitment yielded an improved PaO_2_/F_I_O_2_ in comparison to that achieved with a PEEP level of 10 cm H_2_O before the RM was instigated. This implies that a higher level of oxygenation could be maintained with the same PEEP if an RM is utilized. In some cases (Patients A, B and E), a lower level of post-RM PEEP (5 cm H_2_O), was enough to maintain the PaO_2_/F_I_O_2_ at similar levels to that achieved with a pre-RM PEEP of 10 cm H_2_O.

During the PRM, large fluctuations were clearly noticeable in PaCO_2_ values in all patients. As expected, these coincided with the change in driving pressure that the PRM produced. A reduction in driving pressure increased the PaCO_2_ levels while an increase in driving pressure reduced PaCO_2_ levels. Furthermore, using MRS with a final PEEP of 10 cm H_2_O showed a post RM improvement in PaCO_2_ in all cases. It is highly likely that the above is due to the improved gas exchange following the recruitment of previously de-recruited units.

It is interesting that, in all cases, an increase in ventilator pressure caused a rise in PaCO_2_ initially. This is possibly due to the increasing pressure initially increasing pulmonary dead space, such that for the same minute ventilation, more ventilation was wasted, and consequently, less CO_2_ was eliminated.

The two patients with severe ARDS (Patients B and E) exhibited different responses to the same RM. For example, PaO_2_/F_I_O_2_ dropped significantly post-RM in Patient B for MRS-10, whereas in Patient E PaO_2_/F_I_O_2_ was maintained at a higher level at the end of the maneuver. This reflects the variations that can exist within the ARDS population and provides an example of different pathologies (for example varying distributions of TOPs in ARDS patient [33]), presenting with similar symptoms (in this case similar initial PaO_2_/F_I_O_2_ values). However, as seen in Figures 8 and 9, sufficient oxygen and recruitment could be attained in Patient E with lower inspiratory pressures than those required in Patient B. These results strongly motivate the development of patient-specific ventilation strategies, evaluated by individual PaO_2_ changes, rather than focusing solely on general algorithms.

The implemented model has a number of limitations. The model does not individually consider attributes such as superimposed pressure, the vertical gravitational affect; or surface tension changes on the alveolar and airway walls. Their effects have instead been lumped into the governing equation for the pressure volume relationship of individual alveolar units via the parameter P_ext_, which was determined individually for each alveolar unit within each individual patient during the model configuration stage (see Additional file 1). Effects of overdistension of alveoli have not been modeled and the model also assumes a fixed cardiac output. Therefore, attributes that may be associated with alveolar overdistension, namely, right ventricular impairment, reduced oxygen delivery and increased impact of venous shunting are presently not considered. This explains the smaller than expected rise in PaCO_2_ values [22] observed in our simulation during the recruitment phase of the MRS. Damage to the alveolar-capillary membrane, which can introduce and increase the bacterial and cytokine presence in the systemic circulation [34], and cause further inflammatory responses, is also not currently included in the model. Although the model can determine pulmonary function outcomes (corresponding to respiratory mechanics), information about clinical outcomes associated with RM, such as mortality, cannot be acquired. However, the model does allow for observations to changes in hemodynamic parameters (see Additional file 1 for relevant model equations and Additional file 3 for some examples), and also includes hysteresis (through the inclusion of parameters affecting the alveolar compliance directly, and through time-varying and pressure-dependant changes in the airway resistances (see Additional file 1)), hypoxic pulmonary vasoconstriction, ventilation perfusion mismatch and cyclical collapse-reopening.

The results presented here show the potential of computational simulation to offer an alternative to large-scale clinical trials which have, to date, failed to answer many key questions, such as:

- What level of PEEP should be applied to maintain recruitment in newly opened alveoli?
- What inspiratory pressures need to be maintained to recruit alveoli?
- What are the values and distributions of critical opening times?

A significant limitation with conducting clinical trials is that it is very difficult to compare the results of different studies due to the different cohort of patients included in the trials and other interstudy variations. It should also be noted that there is no evidence of whether the development of atelectasis itself has an adverse affect on the patient. Permissive atelectasis with lower PEEP may be a less deleterious option than risking lung injury using higher PEEP and/or higher tidal volumes in some patients [35]. The optimal PEEP for many RMs is still to be determined and the most recent study in this area by Chiumello *et al*. [30] demonstrated the importance of considering RM timings with applying an RM [30,36].

Apart from the commonly administered RMs considered here, a number of other RMs have recently been proposed that should theoretically improve alveolar function but cannot be tested due to their experimental nature and the lack of patient data that would lend support for their introduction into practice [37-39]. The role of RMs also extends beyond ARDS patients; the administration of RMs has been shown to reduce markers of lung stress in patients following general anesthesia [40-43]. In both cases, computational simulation could play a key role in establishing the potential benefits of RMs and in the design of optimized patient- and disease-specific protocols.

An important question that needs to be addressed in future work in this area is the effect of RMs on the other organs [44]. Mortality linked to ALI and ARDS often involves multiple organ failure, as the disease is not limited to the lungs. RMs also alter the working physiology of surrounding organs, and their impact on the heart and circulation cannot currently be accurately measured [45,46]. Changes in intrathoracic and transpulmonary pressures have a secondary effect of decreasing venous return and cardiac preload, so patients also suffer the additional stress of a reduction in cardiac output during the procedure [47,48]. It is difficult to compare the risks associated with different RMs, although a stepwise RM has been shown to have a smaller effect on cardiac output than the more widely used SI [44,49]. In this study, we focused on the effect of recruitment maneuvers on the pulmonary system. However, we are developing hemodynamic computer simulations of the complete cardiac system that also integrate the pulmonary and systemic circulation in order to better understand these issues. Such research tools could offer an invaluable alternative or complement to time-consuming and expensive clinical trials, and could provide answers to many key unanswered questions about the clinical efficacy of RMs in health and disease.

## Conclusions

Our results indicate that there is significant scope for reducing the high levels of PEEP specified in the MRS without compromising its effectiveness in maintaining adequate oxygenation. More generally, the study highlights the huge potential of computer simulation to assist in evaluating the clinical efficacy of RMs in health and disease, in understanding their modes of operation, and in supporting clinicians in the rational design of improved treatment strategies.

## Key messages

- We present the first application of computer simulation to evaluate the relative efficacy of different recruitment maneuvers with different levels of PEEP for ARDS patients.
- Three recruitment maneuvers (SI, MRS followed by a titrated PEEP and PRM) were applied and evaluated on identical ‘virtual patients’.
- The MRS with titrated PEEP exhibited the most prolonged improvement in oxygenation, but also exposed the alveolar compartments to the highest peak pressures.
- Using the simulator, we were able to establish that there is significant scope for reducing the levels of PEEP specified in the MRS without compromising its effectiveness.

## Additional files

Additional file 1:
**Simulation model description and equations.** The file describes in detail the simulation model employed in the paper. Additional file 2:
**Configuring the simulator to patient data using global optimization and the tabulated configuration results.** The file presents the optimization strategy used in matching the model to the ARDS patient data and the resultant parameter values for the different models tabulated for each patient. Additional file 3:
**Additional figures and experiments.** The file reports the effects on PaO_2_/F_I_O_2_ of changes in hemoglobin levels, cardiac output, and F_I_O_2_ for the MRS-10 RM applied to patient A and presents some further model validation results.

## Abbreviations

ALI: acute lung injury
ARDS: acute respiratory distress syndrome
CO: cardiac output (l min^−1^)
CO_2_: carbon dioxide
CT: computed tomography
F_I_O_2_: fraction of inspired oxygen
Hb: hemoglobin content in blood (gm l^−1^)
IE: inspiratory to expiratory ratio
MRS: maximum recruitment strategy
O_2_: oxygen
P: partial pressure (kPa) for example PO_2_, partial pressure of oxygen
PaCO_2_: partial pressure of carbon dioxide in arterial blood
PaO_2_: partial pressure of oxygen in arterial blood
PaO_2_/F_I_O_2_: ratio of partial pressure of oxygen in arterial blood to fraction of inspired oxygen
PEEP: positive end-expiratory pressure (cm H_2_O)
PEEP_end_: PEEP value at the end of simulation
PEEP_max_: maximum PEEP during a recruitment maneuver
P_peak_: peak alveolar pressure
PRM: prolonged recruitment maneuver
Pv: ventilator pressure
PvCO_2_: partial pressure of carbon dioxide in venous blood
PvO_2_: partial pressure of oxygen in venous blood
Qs/Qt: shunt
RM: recruitment maneuver
RQ: respiratory quotient
SI: sustained inflation
TOP: threshold opening pressure; extra pressure within the collapsed alveolar compartment
VALI: ventilator-associated lung injury
VO_2_: oxygen consumption (ml min^−1^)
VR: respiration rate set by the ventilator (breaths per minute, bpm)
