# Supplementary material for: Evaluation of lung recruitment maneuvers in acute respiratory distress syndrome using computer simulation
Source: Crit Care. 2015 Jan 12;19(1):8. doi: 10.1186/s13054-014-0723-6 (PMC4329196; doi:10.1186/s13054-014-0723-6)
Supplement: Additional file 1: — Simulation model description and equations. The file describes in detail the simulation model employed in the paper. [file 13054_2014_723_MOESM1_ESM.pdf]

Evaluation of lung recruitment maneuvers in Acute Respiratory Distress Syndrome using computer simulation

Anup Das[1], Marc Chikhani[2], Oana Cole[2], Wenfei Wang[1], Tayyba Ali[2], Mainul Haque[2], Declan G. Bates[1] and Jonathan G. Hardman[2]

[1]School of Engineering, University of Warwick, CV4 7AL, UK

[2]School of Medicine, University of Nottingham, NG7 2UH, UK

Corresponding Author: Prof. Declan G. Bates, School of Engineering, University of Warwick, CV4 7AL, UK, Email: D.Bates@warwick.ac.uk

The online data supplement for this paper contains additional material that could not be included in the main text due to space limitations, and is divided into three files. The first file (additional file 1) describes in detail the simulation model employed in the paper. Additional file 2 presents the optimization strategy used in matching the model to the ARDS patient data and the parameter values for the different models. Additional file 3 reports the effects on PaO_2/F_IO_2 of changes in haemoglobin levels, cardiac output, and F_IO_2 for the MRS-10 RM applied to patient A and presents some further model validation results.

Additional File 1: Simulation Model Description and Equations

The model employed in this paper has been developed over the past several years and has been applied and validated on a number of different studies (1-8). The model is organized as a system of several components, each component representing different sections of pulmonary dynamics and blood gas transport, e.g. the transport of air in the mouth, the tidal flow in the airways, the gas exchange in the alveolar compartments and their corresponding capillary compartment, the flow of blood in the arteries, the veins, the cardiovascular compartment, and the gas exchange process in the peripheral tissue compartments. Each component is described as several mass conserving functions and solved as algebraic equations, obtained or approximated from the published literature, experimental data and clinical observations. These equations are solved in series in an iterative manner, so that solving one equation at current time instant (t_k) determines the values of the independent variables in the next equation. At the end of the iteration, the results of the solution of the

final equations determine the independent variables of the first equation for the next iteration.

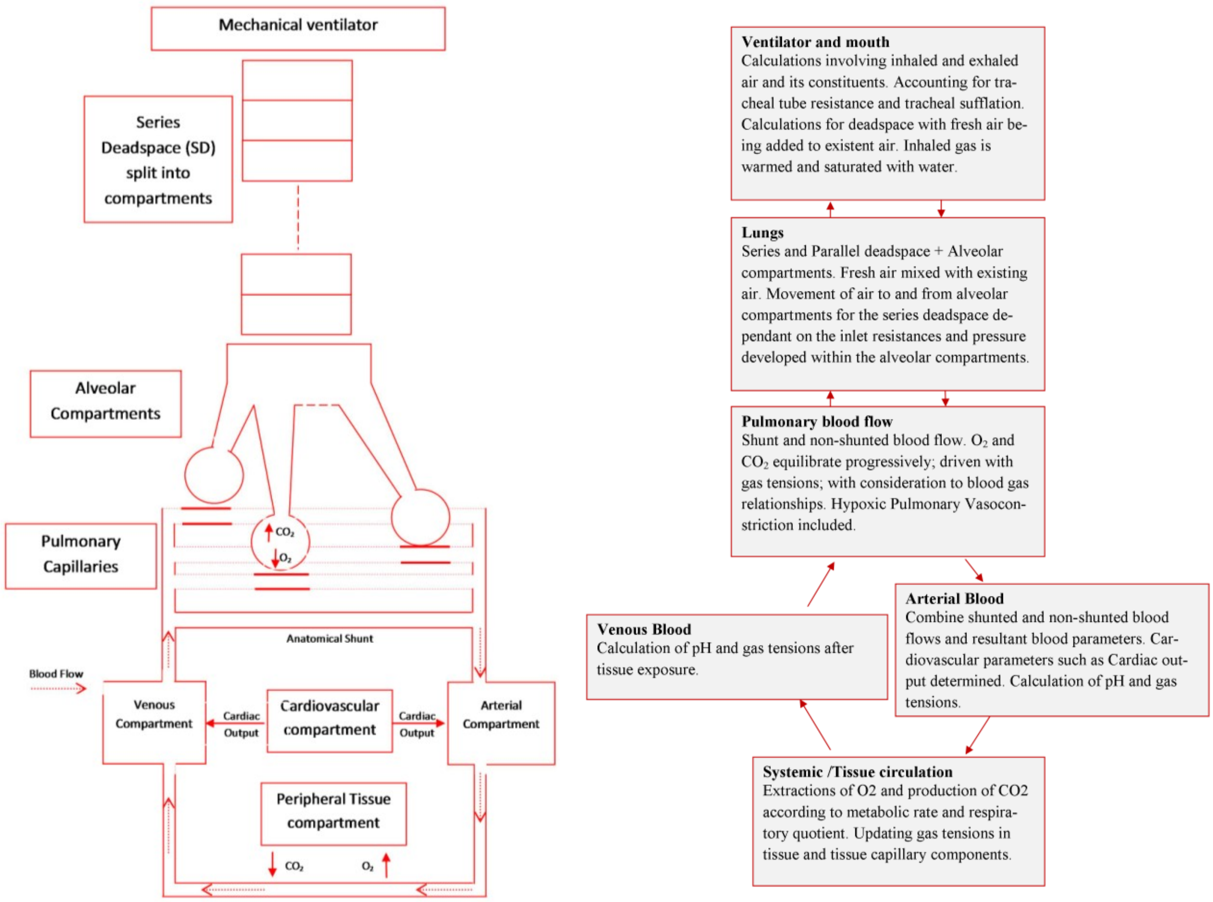

Figure A1: Diagrammatic representation of the model and its main features.

The iterative process continues for a predetermined time, T, representing the total simulation time, with each iteration representing a ‘time slice’ t of real physiological time (set to 30 ms). At the first iteration ($\mathrm{t_k}, \mathrm{k} = 0$), an initial set of independent variables are chosen based on values selected by the user. The user can alter these initial variables to investigate the response of the model or to simulate different pathophysiological conditions. Subsequent iterations ($\mathrm{t_k} = \mathrm{t_{k-1}} + t$) update the model parameters based on the equations below.

The pulmonary model consists of the mechanical ventilation equipment, anatomical and alveolar deadspace, anatomical and alveolar shunts, ventilated alveolar compartments and corresponding perfused capillary compartments. The pressure differential created by the

mechanical ventilator drives the flow of gas through the system. The series deadspace (SD) is located between the mouth and the alveolar compartments and consists of the trachea, bronchi and the bronchioles where no gas exchange occurs. Inhaled gases pass through the SD during inspiration and alveolar gases pass through the SD during expiration. In the model, an SD of volume 60ml is split into 50 stacked layers of equal volumes (N_{SD} = 50). No mixing between the compartments of the SD is assumed.

Any residual alveolar air in the SD at the end of expiration is re-inhaled as inspiration is initiated. This residual air is composed of gases exhaled from both perfused alveolar compartments (normal perfusion) and the parallel deadspace (PD) (alveolar compartments with limited perfusion). Therefore, the size of deadspace (SD and PD) can have a significant effect on the gas composition of the alveolar compartments.

The inhaled air is initially assumed to consist of five gases: oxygen (O_2), nitrogen (N_2), carbon dioxide (CO_2), water vapour (H_2O) and a 5th gas (α) used to model additives such as helium or other anaesthetic gases. During an iteration of the model, the flow (*f*) of air to or from an alveolar compartment *i* at time $\mathrm{t_k}$ is determined by the following equation:

$$f_i(\mathrm{t_k}) = \frac{(p_v(\mathrm{t_k}) - p_i(\mathrm{t_k}))}{(\mathrm{R_u}+\mathrm{R}_{\mathrm{A},\,i})} \qquad \text{for } i = 1, \ldots, N_A \qquad [1]$$

where $p_v(\mathrm{t_k})$ is the pressure supplied by the mechanical ventilator at $(\mathrm{t_k})$, $p_i(\mathrm{t_k})$ is the pressure in the alveolar compartment i at $(\mathrm{t_k})$, $\mathrm{R_u}$ is the constant upper airway resistance and $\mathrm{R}_{\mathrm{A},\,i}$ is the bronchial inlet resistances of the alveolar compartment i. N_A is the total number of alveolar compartments (for the results in this paper, N_A = 100). The total flow of air entering the SD at time $\mathrm{t_k}$ is calculated by

$$f_{SD}(\mathrm{t_k}) = \sum_{i=1}^{N_A} f_i(\mathrm{t_k}) \qquad [2]$$

During the inhaling phase, $f_{SD} \geq 0$, while in the exhaling phase $f_{SD} < 0$.

During gas movement in the SD, the fractions of gases in the layer l of the SD, $\mathrm{F}_{l,}$ $(l = 1, \ldots, N_{SD})$ is updated based on the composition of the total flow, f_{SD}, and the current

composition of $F_{l,\cdot}$. If $f_{SD} \geq 0$, then air starts filling from the top layer (l = 1) to the bottom layer ($l = N_{SD}$); and vice versa for $f_{SD} < 0$.

The volume of gas x, in the ith alveolar compartment ($v_{i,x}$), is given by:

$$v_{i,x}(t_k) = \begin{cases} v_{i,x}(t_{k-1}) - f_i(t_k) \cdot \frac{v_{i,x}(t_{k-1})}{v_i(t_k)} & \textit{Exhaling} \\ v_{i,x}(t_{k-1}) + f_i(t_k) \cdot F_{N_{SD}}(t_k) & \textit{Inhaling} \end{cases} \quad \text{for } i = 1, \ldots, N_A \qquad [3]$$

In [3], x is any of the five gases (O_2, N_2, CO_2, H_2O or α). The total volume of the ith alveolar compartment, v_i is the sum of the volume of the five gases in the compartment.

$$v_i(t_k) = v_{i,O2}(t_k) + v_{i,N2}(t_k) + v_{i,CO2}(t_k) + v_{i,H2O}(t_k) + v_{i,\alpha}(t_k) \qquad [4]$$

For the alveolar compartments, the tension at the centre of the alveolus and at the alveolar capillary border is assumed to be equal. The respiratory system has an intrinsic response to low oxygen levels in blood which is to restrict the blood flow in the pulmonary blood vessels, known as Hypoxic Pulmonary Vasoconstriction (HPV). This is modelled as a simple function, resembling the stimulus response curve suggested by Marshall (9), and is incorporated into the simulator to gradually constrict the blood vessels as a response to low alveolar oxygen tension. The atmospheric pressure is fixed at 101.3kPa and the body temperature is fixed at 37.2°C.

At each t_k, equilibration between the alveolar compartment and the corresponding capillary compartment is achieved iteratively by moving small volumes of each gas between the compartments until the partial pressures of these gases differ by <1% across the alveolar-capillary boundary. The process includes the nonlinear movement of O_2 and CO_2 across the alveolar capillary membrane during equilibration.

In blood, the total O_2 content (C_{O2}) is carried in two forms, as a solution and as oxyhaemoglobin (saturated haemoglobin):

$$C_{O2}(t_k) = S_{O2}(t_{k-1}) \cdot Huf \cdot \text{Hb} + P_{O2}(t_{k-1}) \cdot O_{2sol} \qquad [5]$$

In this equation, S_{O2} is the hemoglobin saturation, *Huf* is the Hufner constant, Hb is the hemoglobin content and O_{2sol} is the O_2 solubility constant. The following pressure-saturation relation, as suggested by (10) to describe the O_2 dissociation curve, is used in this model:

$$S_{O2}(t_k) = \left(\left(\left(P_{O2}^3(t_{k-1}) + 150 \cdot P_{O2}(t_{k-1})\right)^{-1} \times 23400\right) + 1\right)^{-1} \quad [6]$$

S_{O2} is the saturation of the hemoglobin in blood and P_{O2} is the partial pressure of oxygen in the blood. As suggested by (11), P_{O2} has been determined with appropriate correction factors in base excess BE, temperature T and pH (7.5005168 = pressure conversion factor from kPa to mmHg):

$$P_{O2}(t_k) = 7.5006168 \cdot P_{O2}(t_{k-1}) \cdot 10^{[0.48(pH(t_{k-1})-7.4)-0.024(T-37)-0.0013 \cdot BE]} \quad [7]$$

The CO_2 content of the blood (C_{CO2}) is deduced from the plasma CO_2 content ($C_{CO2plasma}$) (12) by the following equation:

$$C_{CO2}(t_k) = C_{CO2plasma}(t_{k-1}) \cdot \left[1 - \frac{0.0289 \cdot Hb}{(3.352 - 0.456 . S_{O2}(t_k)) \cdot (8.142 - pH(t_{k-1}))}\right] \quad [8]$$

where S_{O2} is the O_2 saturation, Hb is the hemoglobin concentration and pH is the blood pH level. The coefficients were determined as a standardized solution to the McHardy version of Visser's equation (13), by iteratively finding the best fit values to a given set of clinical data. The value of $C_{CO2plasma}$ is deduced using the Henderson-Hasselbach logarithmic equation for plasma C_{CO2} (14).

$$C_{CO2plasma}(t_k) = 2.226 \cdot s_{CO2} \cdot P_{CO2}(t_{k-1}) \left(1 + 10^{(pH(t_{k-1}) - pK')}\right) \quad [9]$$

where s_{CO2} is the plasma CO_2 solubility coefficient and pK' is the apparent pK (acid dissociation constant of the CO_2 bicarbonate relationship). P_{CO2} is the partial pressure of CO_2 in plasma and '2.226' refers to the conversion factor from miliMoles per liter to ml/100ml. (14) gives the equations for s_{CO2} and pK' as:

$$s_{CO2} = 0.0307 + 0.0057 \cdot (37 - T) + 0.00002 \cdot (37 - T)^2 \quad [10]$$

$$pK' = 6.086 + 0.042 \cdot (7.4 - pH(t_{k-1})) + (38 - T) \cdot \left(0.00472 + \left(0.00139 - (7.4 - pH(t_{k-1}))\right)\right) \quad [11]$$

$P_{CO2}(t_k)$ is determined by incorporating the standard Henry's law and the s_{CO2} (the CO_2 solubility coefficient above). For pH calculation, the Henderson Hasselbach and the Van Slyke equation (15) are combined. Below is the derivation of the relevant equation. The Henderson-Hasselbach equation (governed by the mass action equation (acid dissociation)) states that:

$$pH = pK + \log\left(\frac{bicarbonate\ concentration}{carbonic\ acid\ concentration}\right) \quad [12]$$

Substituting pK=6.1 (under normal conditions) and the denominator $(0.225 \cdot P_{CO2})$ (acid concentration being a function of CO_2 solubility constant 0.225 and P_{CO2} (in kPa)) gives:

$$pH(t_k) = 6.1 + \log\left(\frac{HCO_3(t_{k-1})}{0.225 \cdot P_{CO2(t_k)}}\right) \quad [13]$$

For a given pH, base excess (BE), and hemoglobin content (Hb), HCO_3 is calculated using the Van-Slyke equation, as given by (15):

$$HCO_3(t_k) = \left((2.3 \times Hb + 7.7) \times (pH(t_k) - 7.4)\right) + \frac{BE}{(1 - 0.023 \times Hb)} + 24.4 \quad [14]$$

The capillary blood is mixed with arterial blood using the equation below which considers the anatomical shunt (Sh) with the venous blood content of gas x ($C_{v,x}$), the non-shunted blood content from the pulmonary capillaries ($C_{cap,x}$), arterial blood content ($C_{a,x}$), the arterial volume (v_a) and the cardiac output (CO).

$$C_{a,x}(t_k) = \frac{CO(t_k) \cdot \left(Sh \cdot C_{v,x}(t_k) + (1 - Sh) \cdot C_{cap,x}(t_k)\right) + C_{a,x}(t_k) \cdot (v_a(t_k) - CO(t_k))}{v_a(t_k)} \quad [15]$$

The peripheral tissue model consists of a single tissue compartment, acting between the peripheral capillary and the *active* tissue (undergoing respiration to produce energy). The consumed O_2 (V_{O2}) is removed and the produced CO_2 (V_{CO2}) is added to this tissue compartment. Similarly to alveolar equilibration, peripheral capillary gas partial pressures

reach equilibrium with the tissue compartment partial pressures, with respect to the nonlinear movement of O_2 and CO_2. Metabolic production of acids, other than carbonic acid via CO_2 production, is not modeled. After peripheral tissue equilibration of gases, the venous calculations of partial pressures, concentrations and pH calculations are done using comparable equations as above.

A simple equation of renal compensation for acid base disturbance is incorporated. The base excess (BE) of blood under normal conditions is zero. BE increases by 0.1 per time slice if pH falls below 7.36 (to compensate for acidosis) and decreases by 0.1 per time slice if pH rises above 7.4 (under alkalosis).

The shunt fraction (Q_S/Q_T) in the model is calculated as:

$$Q_S/Q_T = (C_{cO2}(t_k) - C_{aO2}(t_k))/(C_{cO2}(t_k) - C_{vO2}(t_k)) \quad [16]$$

where the end capillary oxygen content (C_{cO2}), the arterial oxygen content (C_{aO2}) and the mixed venous oxygen (C_{vO2}) content can also be obtained from the model. The total compliance (E_{dyn}) of the lung in the model is calculated using the standard equation:

$$E_{dyn}(t_k) = (V_{max}(t_k) - V_{min}(t_k))/(P_{max}(t_k) - P_{min}(t_k)) \quad [17]$$

where the End-Inspiratory Lung Volume (V_{max}), End-Expiratory Lung Volume (V_{min}), maximum pressure in lung (P_{max}) and the minimum pressure in lung (P_{min}) are obtained directly from the model at the end of every breath.

The simulated patient is assumed to be under complete mechanical ventilation. Consequently, the effects of ventilatory autoregulation by the patient have not been incorporated into the models.

Each alveolar compartment has a unique and configurable alveolar compliance, alveolar inlet resistance, vascular resistance, extrinsic (interstitial) pressure and threshold opening pressure. For the ith compartment of N alveolar compartments, the pressure p_i is determined by:

$$p_i(\mathrm{t_k}) = \begin{cases} S_i(v_i(\mathrm{t_k}) - V_c)^2 - P_{ext,i} & v_i(\mathrm{t_k}) > 0 \\ 0 & \end{cases} \quad \text{for } i = 1, \dots, N_A \qquad [18]$$

where

$$S_i = k_i {N_A}^2/200000 \quad \text{and} \quad V_c = 0.2V_{FRC}/N_A$$

Equation [18] determines the alveolar pressure p_i (as the pressure above atmospheric in cmH_2O) for the ith compartment of *N* number of alveolar compartments for the given volume of alveolar compartment, $v_i(t)$ in milliliters. The alveolar compartments are arranged in parallel and interact with the series deadspace with respect to the movement of gases. The flow of air into the alveolar compartments is achieved by a positive pressure provided by the ventilator and the air moves along the pressure gradient. The equation models the behavior of the intact lung / chest-wall complex. The use of the square of the difference between v_i and *Vc* causes alveolar pressure to increase at volumes below *Vc*, leading to exhalation and a tendency to "snap shut" (mathematical note: the pressure with respect to volume is thus a U-shaped curve).

P_{ext} (per alveolar unit, in cmH_2O) represents the *effective net pressure* generated by the sum of the effects of factors *outside each alveolus* that act to distend that alveolus; positive components include the outward pull of the chest wall, and negative effects include the compressive effect of interstitial fluid in the alveolar wall. Incorporating P_{ext} in the model allows us to replicate the situation of alveolar units that have less structural support or that have interstitial oedema, and thus have a greater tendency to collapse. A negative value of P_{ext} indicates a scenario where there is compression from outside the alveolus causing collapse. The parameter S_i is a scalar that determines the intra-alveolar pressure for a given volume (with respect to a constant collapsing volume V_c) and is dependent on the parameter k. The units of S_i are cmH_2O ml^{-2}. Finally, V_c is defined as a "constant collapsing volume" at which the alveolus tends to empty (through Laplace effects) and represents a fundamental mechanical property of tissue and surfactant. V_{FRC} is the resting volume of the lung (assumed to be 3 litres).

The effect of the three parameters on the volume–pressure relationship of the alveolar compartments can be observed in the following Figure A2.

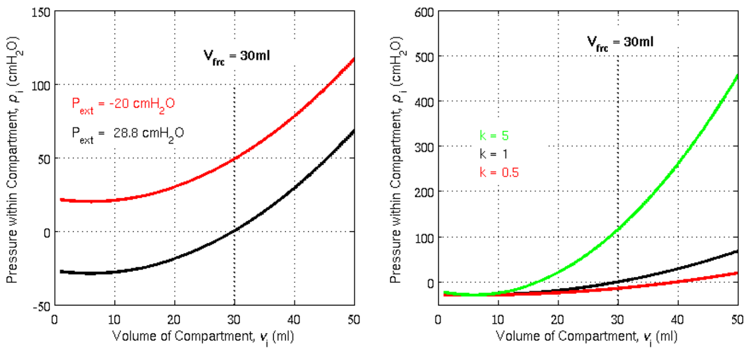

Figure A2: The effect of varying the parameters of Equation [18] on the pressure volume relationship of the model.

For a healthy lung at the end of the expiration, the ventilator pressure would return to zero above atmospheric (resulting in the tracheal pressure also being equal to zero). The nominal values for ($P_{ext,i}$, S_i) have been determined such that at the end of expiration, the alveolar pressure within the compartment is also equal to zero, i.e. at 30 ml, the individual compartments are at rest and consequently the total resting volume of the lung is 3 liters.

We consider each of the three parameters mentioned above ($P_{ext,i}$, S_i) to be different yet essential components for representing a diseased lung that affect the volume pressure relationship of the alveolar compartments. For example, for a given volume v_i, increasing S_i increases the corresponding alveolar pressure of the alveolar compartment. When compared to another compartment with a lower S_i, a larger pressure from the mechanical ventilator would be needed to drive air into the compartment; thus effectively the compartment will be behaving as a stiffer lung unit.

Decreasing $P_{ext,i}$ increases the alveolar pressure such that the pressure gradient (especially during exhaling) forces the air out of the alveolar compartment until the volume of the compartment collapses (v_i = 0 ml). Note that, in effect, the parameters are influencing the resting volume of the compartments (when the alveolar pressure, p_i, is equal to zero). If $p_i <$ 0 cmH$_2$O, the pressure gradient will cause the flow into the alveolar compartment (as ventilator pressure will always be ≥ 0 cmH$_2$O) until p_i reaches 0 cmH$_2$O.

In the model, the airway resistance R_{aw} is determined by the following equation for N parallel compartments:

$$\frac{1}{R_{aw}} = \frac{1}{R_{B,1}} + \frac{1}{R_{B,2}} + \cdots + \frac{1}{R_{B,N_A}}, \text{ for } i = 1, \dots, N_A \quad [19]$$

where $R_{B,i}$ is the bronchial inlet resistance of the ith compartment, which is defined by:

$$R_{B,i} = m_i R_{B0}$$

where R_{B0} corresponds to the default bronchial inlet resistance of an alveolar compartment. R_{B0} is set to $1 \times 10^{-5} \cdot \mathrm{N}$ (the inlet resistance is higher for a model with more compartments as the volume of each compartment decreases) for a healthy lung, giving a resistance of 0.001 kpa per ml per minute for 100 compartments. m_i is a coefficient of the airway resistance, representing a dynamic change in airway resistance and is determined by the equation:

$$m_i = \begin{cases} 1, & \mathrm{t}_{\mathrm{o},i} \leq 0 \\ 10^{10}, & \mathrm{t}_{\mathrm{o},i} > 0 \end{cases} \text{ for } i = 1, \dots, N_A \quad [20]$$

where,

$$\mathrm{t}_{\mathrm{o},i} = \begin{cases} \mathrm{t}_{\mathrm{o},i} - t, & p_{trachea} \geq \mathrm{TOP}_i \\ \tau_{c,i}, & p_{trachea} < \mathrm{TOP}_i \end{cases} \text{ for } i = 1, \dots, N_A \quad [21]$$

$p_{trachea}$ is the pressure in the trachea and TOP_i is a value between 5 and 50 $\mathrm{cmH_2O}$ for the ith alveolar compartmen. Additionally, a threshold opening pressure (TOP) at low lung volumes needs to be attained for a collapsed alveolar unit to open. Recruitment is a time dependent process, with different airways recruiting at different times, once the threshold pressure has been achieved (16-17). The equations within the model are solved iteratively as a discretised system. Each iteration represents a physiological time slice of *t* (10 ms). The time dependant recruitment phenomenon is achieved in the model by the introduction of a parameter $\mathrm{t_o}$. For collapsed compartments, $\mathrm{t_o}$ is set to τ_c which represents the time it could take for collapsed alveoli to open after a threshold pressure is reached. Once $p_{trachea} \geq \mathrm{TOP_i}$ is satisfied, the counter $\mathrm{t_o}$ decrements during every iteration, and triggers the opening of the airway (m_i= 1) as $\mathrm{t_o} \leq 0$. Otherwise m_i is set to a high value (10^{10}) to represent a collapsed airway. We based the range of values for TOP used in these simulations on the work done by Crotti and collaborators (18).

N_A (the number of alveolar compartments) is fixed and set by the user (i.e. they do not change during a simulation). Therefore, during a simulation, m_i, chiefly represents the relatively small changes in inlet resistance during tidal ventilation. Furthermore, R_{B0} are also preset and fixed, and do not change during the simulation. The only change in airway resistance which is dynamic is m_i which is dependent on the volume v_i at time($\mathrm{t_k}$).

Finally, the pulmonary vascular resistance PVR is determined by

$$\frac{1}{PVR} = \frac{1}{R_{V,1}} + \frac{1}{R_{V,2}} + \cdots + \frac{1}{R_{V,N_A}}, \text{ for } i = 1, \dots, N_A \quad [23]$$

where the resistance for each compartment $R_{V,i}$ is defined as

$$R_{V,i} = \delta_{Vi} R_{V0} \quad [24]$$

R_{V0} is the default vascular resistance for the compartment with a value of $160 \cdot N_A$ dynes s cm^{-5} min^{-1}, and δ_{Vi} is the vascular resistance coefficient, used to implement the effect of Hypoxic Pulmonary Vasoconstriction.

The net effect of these components of the simulation is that the defining, clinical features of ARDS may be observed in the model: alveolar gas-trapping (with intrinsic PEEP), collapse-reopening of alveoli (with gradual reabsorption of trapped gas if re-opening does not occur), limitation of expiratory flow etc.

References

1. Hardman J, Aitkenhead A. Estimation of alveolar deadspace fraction using arterial and end-tidal CO2: a factor analysis using a physiological simulation. *Anaesthesia and intensive care*. 1999;27(5):452.
2. Hardman J, Bedforth N. Estimating venous admixture using a physiological simulator. *British journal of anaesthesia*. 1999;82(3):346-9.
3. Hardman J, Bedforth N, Ahmed A, et al. A physiology simulator: validation of its respiratory components and its ability to predict the patient's response to changes in mechanical ventilation. *British journal of anaesthesia*. 1998;81(3):327-32.
4. Hardman JG, Aitkenhead AR. Validation of an original mathematical model of CO2 elimination and dead space ventilation. *Anesthesia & Analgesia*. 2003;97(6):1840-5.
5. Hardman J, Wills J. The development of hypoxaemia during apnoea in children: a computational modelling investigation. *British journal of anaesthesia*. 2006;97(4):564-70.
6. Das A, Gao Z, Menon P, Hardman J, Bates D. A systems engineering approach to validation of a pulmonary physiology simulator for clinical applications. *Journal of The Royal Society Interface* 2011; 8: 44-55.
7. McCahon R, Columb M, Mahajan R, Hardman J. Validation and application of a high-fidelity, computational model of acute respiratory distress syndrome to the examination of the indices of oxygenation at constant lung-state. *British journal of anaesthesia* 2008; 101: 358-365.
8. Al-otaibi H, Hardman JG. Prediction of arterial oxygen tension:validation of a novel formula. *Am J Resp Crit Care* 2010 ; 182(3) : 435-436.
9. Marshall BE, Clarke WR, Costarino AT, Chen L, et al. The dose-response relationship for hypoxic pulmonary vasoconstriction. *Respir Physiol.* 1994 May;96(2-3):231-47.

10. Severinghaus JW. Simple, accurate equations for human blood O2 dissociation computations. *J Appl Physiol Respir Environ Exerc Physiol*. 1979 Mar;46(3):599-602.

11. Severinghaus JW. Blood gas calculator. J Appl Physiol. 1966 May;21(3):1108-16.

12. Douglas AR, Jones NL, Reed JW. Calculation of whole blood CO2 content. *J Appl Physiol* 1988 Jul;65(1):473-7.

13. McHardy GJ. The relationship between the differences in pressure and content of carbon dioxide in arterial and venous blood. *Clin Sci.* 1967 Apr;32(2):299-309.

14. Kelman GR, Nunn JF. Nomograms for correction of blood Po2, Pco2, pH, and base excess for time and temperature. *J Appl Physiol*. 1966 Sep;21(5):1484-90.

15. Siggaard-Andersen O. The van Slyke equation. *Scand J Clin Lab Invest Suppl*. 1977;146:15-20.

16. Hickling KG. The pressure-volume curve is greatly modified by recruitment. A mathematical model of ARDS lungs. *Am J Respir Crit Care Med*. 1998 Jul;158(1):194-202.

17. Bates JH, Irvin CG. Time dependence of recruitment and derecruitment in the lung: a theoretical model. *J Appl Physiol* 2002; 93(2):705-13.

18. Crotti S, Mascheroni D, Caironi P, Pelosi P et al. Recruitment and derecruitment during acute respiratory failure: a clinical study. *Am J Respir Crit Care Med* 2001;164(1):131-40.
