# Supplementary material for: Evaluation of lung recruitment maneuvers in acute respiratory distress syndrome using computer simulation
Source: Crit Care. 2015 Jan 12;19(1):8. doi: 10.1186/s13054-014-0723-6 (PMC4329196; doi:10.1186/s13054-014-0723-6)
Supplement: Additional file 2: — Configuring the simulator to patient data using global optimization and the tabulated configuration results. The file presents the optimization strategy used in matching the model to the ARDS patient data and the resultant parameter values for the different models tabulated for each patient. [file 13054_2014_723_MOESM2_ESM.pdf]

Evaluation of lung recruitment maneuvers in Acute Respiratory Distress Syndrome using computer simulation

Anup Das[1], Marc Chikhani[2], Oana Cole[2], Wenfei Wang[1], Tayyba Ali[2], Mainul Haque[2], Declan G. Bates[1] and Jonathan G. Hardman[2]

[1]School of Engineering, University of Warwick, CV4 7AL, UK

[2]School of Medicine, University of Nottingham, NG7 2UH, UK

Corresponding Author: Prof. Declan G. Bates, School of Engineering, University of Warwick, CV4 7AL, UK, Email: D.Bates@warwick.ac.uk

The online data supplement for this paper contains additional material that could not be included in the main text due to space limitations, and is divided into three files. The first file (additional file 1) describes in detail the simulation model employed in the paper. Additional file 2 presents the optimization strategy used in matching the model to the ARDS patient data and the parameter values for the different models. Additional file 3 reports the effects on PaO_2/F_IO_2 of changes in haemoglobin levels, cardiac output, and F_IO_2 for the MRS-10 RM applied to patient A and presents some further model validation results.

Additional File 2: Configuring the Simulator to Patient Data using Global Optimization and the tabulated configuration results

The model was configured to fit data from individual ARDS patients in two stages. In the first stage, the model was matched to data reported by Nirmalan and colleagues (1), which listed measurements taken from 10 patients treated for ARDS. The data consisted of arterial and mixed venous blood gas values and cardiac output estimations for each patient, listing the following measurements: hemoglobin content in blood in g dl-1 (Hb), cardiac output in ml min-1(CO), partial pressure of oxygen in arterial blood (PaO_2), partial pressure of oxygen in mixed venous blood (PvO_2), partial pressure of carbon dioxide in mixed venous blood ($PvCO_2$), shunt fraction (QS/QT) and F_IO_2. To simulate an individual patient, one set of data (a row from Table 1 of the supplementary material given with (1)) was selected and the values of Hb, CO and F_IO_2 were fixed as given. We then used an optimization-based methodology (see below) to find the model configuration that would generate outputs that

most closely matched the clinical data available. The model parameters to be optimized for each of the 100 alveolar compartments were P_{ext}, k and "TOP", representing the extrinsic pressure acting on an alveolar compartment, the stiffness of the compartment and the threshold opening pressure, respectively (see Table 1 below). As values for respiratory quotient (RQ), rate of breathing (VR), total oxygen consumption (VO_2), and IE ratio used were not reported in the original study these settings were also simultaneously optimized to provide the best possible fit to the data. The PEEP and peak inspiratory pressure (P_v) were set to 5 cm H_2O and 15 cm H_2O respectively, to prepare the "virtual patients" for the RM trials to be performed later.

The data obtained from Nirmalan (1) contain only static measurements and thus do not provide information concerning dynamic processes such as recruitment. Therefore, after a lung configuration was determined using the methodology described above, a second round of optimization was done to determine the value of τ_c for each alveolar compartment so as to best fit the dynamic recruitment data provided by Chiumello and colleagues (2). The authors of this study report blood gas measurements in ARDS patients over a 60-minute period as a result of step changes in PEEP. We replicated the above-mentioned trial in our simulator through a simple PEEP maneuver consisting of two stages, an increase in PEEP from 5 cm H_2O to 15 cm H_2O followed by a decrease in PEEP from 15 cm H2O to 5 cm H_2O. Each stage lasted for a period of 60 minutes. All relevant parameters concerning ventilation and hemodynamics are determined as follows: the model lung parameters i.e. P_{ext}, k and "TOP", are configured as determined by the initial optimization procedure. The ventilator parameters F_IO_2, RR, P_v and IE are set to data reported in (2). The CO, VO_2 and RQ are then adjusted to fit the initial value of PaO_2 (73 mmHg) given in the data obtained from (2). Finally, an optimization-based search is conducted to find values of τ_c for all the 100 compartments of the model, such that the least-squares difference between the PaO_2 output of the model and the PaO_2 values reported in (2) is minimized.

Details of the optimization based methodology utilized to find an *in silico* lung configuration that would find the best fit to patient measurements recorded in the data are as follows. In stage 1, the optimization problem was formulated to find the minimum value of an objective function J in the equation below:

$$\min_x J = \sqrt[2]{\sum_{i=1}^{4} r_i^2} \quad where\; r_i = \frac{y_i - y_i'}{y_i'} \qquad [26]$$

where y = [PaO_2, PvO_2, $PvCO_2$, Q_S/Q_T] are the model outputs and y' = [PaO_2', PvO_2', $PvCO_2$', Q_S/Q_T'] are the measurements obtained from the data.

The optimization parameters (x) in stage 1, their sizes, ranges and units are summarized in Table S1. The value of τ_c [21] is set randomly for each compartment between 0.001 and 10 minutes at stage 1.

Stage 2 of the matching process required a search for the optimal distribution of the parameter τ_c in Equation [21] that minimizes the difference between model output (PaO_2) and the observed change in O_2 in the dynamic data (PaO_2') given in Figures 1 and 2 (2). The change in O_2 was observed in response to a stimulus provided by modifying the PEEP from 5 cm H_2O to 15 cm H_2O and then back to 5 cm H_2O. The total simulation period was 110 minutes, with PaO_2 recorded at specific intervals corresponding to the intervals recorded in the data. Equation [26] is thus again utilized with y = [PaO_2] and y' = [PaO_2'] and the optimization parameter (x) consisting solely of the vector τ_c with an upper and lower bound of 10 and 0.001mins, respectively.

A genetic algorithm (GA) was employed for both optimization procedures. GA's are general purpose stochastic search and optimization procedures based on evolutionary concepts (3) such as selection, mutation, recombination etc. In GA's, a randomly selected population of candidates (1st generation) undergoes a repetitive process of reproduction, where selection is based on the value of the objective function (also called the fitness). Every generation, the best candidates from the previous generation (elitism) and candidates obtained through mutation and crossover, recombine to form a new population. The average fitness of the individuals in the population is expected to increase as strong individuals are protected and combined with one other and weak individuals are discarded. Due to their ease of application in problems with large and small parameter search spaces, GA's are a popular search and optimization technique. With their probabilistic and parallel nature, GA's are generally capable of converging to the global optimum even in highly non-convex parameter spaces –

convergence can be slow, however, and global algorithms like GA's typically require much longer computation times than local gradient-based methods (4). To speed up the optimization process, a parallelised computer code implementation of a genetic algorithm was employed in this study. The cost function evaluation process associated with a population can be accelerated hugely by distributing the tasks to multiprocessors (multiple cores and/or multiple machines). High performance computing facilities available at the University of Warwick were configured and implemented to run the parallel computing jobs.

The matching process was performed under Matlab 2011a using the Global Optimization Toolbox and Parallel Computing Toolbox. An adaptive termination strategy, which allows the optimization algorithm to run as long as necessary, was applied for each case to ensure the global optimal was reached.

References

1. Nirmalan M, Willard T, Columb MO, Nightingale P. Effect of changes in arterial-mixed venous oxygen content difference (C(a-v)O2) on indices of pulmonary oxygen transfer in a model ARDS lung. *Br J Anaesth* 2001; 86: 477-485.
2. Chiumello D, Coppola S, Froio S, Mietto C, et al. Time to reach a new steady state after changes of positive end expiratory pressure. *Intensive Care Med*. 2013; 39(8):1377-85.
3. Goldberg DE. The design of innovation : lessons from and for competent genetic algorithms. Boston: Kluwer Academic Publishers; 2002.
4. Fleming PJ, Purshouse RC. Evolutionary algorithms in control systems engineering: a survey. *Control engineering practice*. 2002;10(11):1223-41.

Configured values of parameters

Table A1: Optimization parameters and their ranges

Parameters	Size	Range	Units
TOP, see [21]	100	5, 40	cm H_2O
k, see [18]	100	10^{-0}, $10^{0.5}$	-
P_{ext}, see [18]	100	-15, 28	cm H_2O
VR	1	12, 30	breaths min^{-1}
IE	1	0.25,0.50	-
RQ	1	0.6-0.9	-
VO_2	1	200, 300	ml min^{-1}

Definition of Abbreviations: TOP = Threshold Opening Pressure, P_{ext} = Extrinsic Pressure see [15], k = Stiffness coefficient, see [16], VR = Ventilation Rate, IE: Inspiratory to Expiratory Ratio, RQ = Respiratory Quotient, VO_2 = Oxygen Consumption

Table A2: Results of data matching, static data, Patient A,
i = alveolar compartment number

i	TOP	k	P_{ext}		i	TOP	k			i	TOP	k	P_{ext}
1	11.63	0.31	28.80		35	24.45	0.06	21.26		69	41.53	0.34	-0.06
2	33.35	0.19	-11.54		36	31.52	0.41	-13.42		70	35.44	0.02	2.71
3	28.32	0.09	-9.98		37	13.68	0.23	28.16		71	35.26	0.14	-9.29
4	10.83	0.35	24.50		38	38.34	0.23	-14.39		72	41.23	0.14	7.67
5	32.57	0.46	5.52		39	36.19	0.41	-11.69		73	29.99	0.05	-7.73
6	45.00	0.00	24.65		40	26.07	0.24	9.52		74	42.43	0.29	-5.44
7	36.03	0.18	15.73		41	38.22	0.16	-14.77		75	10.94	0.12	18.78
8	43.31	0.50	-11.33		42	31.57	0.27	-11.27		76	25.46	0.06	21.25
9	33.36	0.36	-2.76		43	20.66	0.29	0.97		77	42.84	0.01	8.43
10	38.71	0.33	22.18		44	12.01	0.26	17.63		78	29.85	0.00	16.77
11	19.52	0.24	7.81		45	35.81	0.10	5.69		79	13.97	0.12	11.57
12	40.28	0.23	-13.76		46	30.68	0.46	-0.11		80	29.53	0.02	6.13
13	26.45	0.49	-14.16		47	10.47	0.22	13.34		81	30.43	0.00	3.44
14	27.89	0.28	9.91		48	34.78	0.34	-9.53		82	16.99	0.34	0.14
15	32.45	0.37	-13.79		49	32.66	0.49	-8.68		83	34.19	0.05	23.56
16	27.69	0.26	-13.15		50	44.36	0.19	10.26		84	31.99	0.03	17.11
17	42.09	0.31	-13.35		51	36.71	0.32	16.82		85	32.03	0.23	12.88
18	28.20	0.30	4.79		52	36.40	0.07	8.03		86	14.07	0.01	11.98
19	24.88	0.37	-8.33		53	37.02	0.07	27.40		87	15.15	0.12	-6.79
20	23.34	0.13	-11.81		54	34.42	0.06	7.96		88	43.76	0.18	-0.02
21	37.65	0.25	-5.23		55	19.49	0.14	3.17		89	41.47	0.26	14.06
22	11.47	0.00	25.29		56	38.44	0.46	-7.55		90	27.37	0.13	16.60
23	26.74	0.38	6.10		57	42.92	0.34	-10.36		91	30.55	0.16	-5.98
24	45.00	0.17	-15.00		58	25.53	0.31	2.94		92	26.06	0.00	28.80
25	34.79	0.47	-7.71		59	14.92	0.14	14.85		93	30.77	0.31	-6.10
26	26.16	0.05	27.70		60	21.03	0.04	23.21		94	33.24	0.03	6.26
27	34.77	0.42	-11.28		61	43.46	0.28	15.41		95	36.52	0.45	-9.53
28	39.50	0.10	18.69		62	37.15	0.39	-3.04		96	24.15	0.26	12.65
29	16.16	0.21	-5.61		63	35.11	0.24	12.01		97	32.00	0.01	5.89
30	37.11	0.19	-11.51		64	13.60	0.03	28.48		98	23.00	0.26	-1.49
31	44.40	0.32	-6.30		65	31.59	0.05	16.05		99	13.25	0.02	-0.07
32	27.57	0.02	-13.33		66	17.39	0.09	15.85		100	28.78	0.04	15.71
33	40.07	0.05	13.32		67	45.00	0.34	-11.23					
34	38.42	0.15	14.55		68	42.07	0.13	-10.20					

Table A3: Results of data matching, static data, Patient B

i = alveolar compartment number

i	TOP	k	P_{ext}		i	TOP	k			i	TOP	k	P_{ext}
1	45.00	0.28	-15.00		35	16.60	0.26	-0.12		69	32.95	0.12	9.54
2	10.00	0.28	27.69		36	33.19	0.00	7.17		70	44.36	0.00	14.06
3	21.90	0.25	-13.66		37	31.87	0.50	-15.00		71	40.59	0.00	28.80
4	16.24	0.12	-10.37		38	42.88	0.34	-3.11		72	24.13	0.00	28.80
5	10.00	0.10	18.18		39	27.50	0.18	28.80		73	43.05	0.29	-7.35
6	19.91	0.24	12.62		40	25.71	0.26	-15.00		74	14.12	0.25	-9.37
7	33.50	0.43	-13.72		41	33.50	0.04	27.35		75	10.00	0.26	-13.78
8	45.00	0.50	-7.93		42	17.30	0.00	26.94		76	27.50	0.22	6.90
9	45.00	0.20	-13.75		43	32.98	0.18	6.26		77	26.20	0.08	27.15
10	10.00	0.00	18.16		44	12.36	0.04	10.90		78	33.09	0.13	6.90
11	45.00	0.04	-13.27		45	26.97	0.01	-15.00		79	24.07	0.47	-9.48
12	16.54	0.50	-12.28		46	24.61	0.00	28.08		80	45.00	0.00	26.85
13	26.40	0.00	-3.70		47	39.59	0.12	1.28		81	37.66	0.10	8.04
14	40.01	0.49	-8.69		48	45.00	0.00	9.05		82	43.02	0.04	14.57
15	23.91	0.28	-15.00		49	32.01	0.03	7.81		83	44.30	0.07	11.27
16	25.33	0.48	-8.26		50	38.15	0.43	-9.18		84	13.42	0.39	15.24
17	45.00	0.00	0.90		51	28.85	0.24	6.23		85	39.69	0.38	13.42
18	45.00	0.20	16.45		52	27.50	0.18	11.99		86	42.11	0.11	-8.22
19	45.00	0.00	1.45		53	35.01	0.05	22.31		87	23.23	0.12	-7.37
20	39.25	0.50	-14.46		54	27.38	0.10	-1.26		88	31.27	0.42	-2.90
21	10.00	0.46	-9.22		55	45.00	0.05	28.54		89	10.00	0.23	2.20
22	40.05	0.25	-8.09		56	38.21	0.07	8.30		90	10.00	0.20	6.36
23	10.15	0.00	1.87		57	34.00	0.50	-15.00		91	29.34	0.20	6.90
24	36.06	0.13	-15.00		58	23.55	0.19	-12.87		92	40.14	0.17	28.80
25	45.00	0.06	-2.84		59	15.32	0.03	14.29		93	45.00	0.17	14.77
26	40.22	0.29	-13.29		60	26.84	0.18	-8.85		94	15.83	0.26	6.93
27	30.56	0.18	12.95		61	28.07	0.04	-15.00		95	30.78	0.27	-8.43
28	32.64	0.43	1.18		62	37.17	0.00	9.59		96	26.06	0.50	-15.00
29	13.72	0.00	16.61		63	45.00	0.31	0.81		97	31.84	0.37	19.63
30	43.05	0.16	15.48		64	45.00	0.16	16.16		98	40.18	0.20	15.99
31	10.00	0.04	-15.00		65	29.00	0.33	-15.00		99	10.00	0.41	-15.00
32	10.54	0.00	-13.86		66	10.00	0.00	17.23		100	42.21	0.30	8.65
33	13.33	0.48	-9.33		67	37.74	0.03	-15.00					
34	10.00	0.20	-0.10		68	27.50	0.21	8.35					

Table A4: Results of data matching, static data, Patient C

i = alveolar compartment number

i	TOP	k	P_{ext}		i	TOP	k			i	TOP	k	P_{ext}
1	21.43	0.41	-5.66		35	23.07	0.08	-11.38		69	45.00	0.09	19.33
2	32.13	0.48	15.15		36	36.57	0.34	11.00		70	42.26	0.47	12.36
3	12.11	0.49	-12.81		37	45.00	0.02	23.56		71	42.46	0.12	12.85
4	35.35	0.13	-15.00		38	42.14	0.43	17.60		72	23.29	0.37	-2.45
5	38.25	0.35	9.89		39	31.70	0.44	21.04		73	30.03	0.37	19.44
6	43.14	0.26	-14.20		40	42.76	0.28	15.73		74	12.61	0.44	-13.09
7	42.50	0.49	1.98		41	35.44	0.49	14.03		75	30.43	0.13	24.35
8	25.03	0.48	5.68		42	32.13	0.14	27.82		76	42.03	0.43	17.12
9	34.59	0.30	0.92		43	34.26	0.37	19.28		77	32.41	0.49	27.26
10	32.42	0.42	18.15		44	35.04	0.00	24.66		78	19.31	0.09	18.18
11	22.38	0.33	-13.63		45	28.08	0.41	28.32		79	42.68	0.12	17.32
12	27.11	0.46	-10.29		46	33.84	0.41	25.11		80	44.68	0.00	19.00
13	25.49	0.47	13.11		47	40.30	0.31	-13.12		81	32.43	0.36	-15.00
14	37.02	0.47	15.62		48	20.10	0.44	21.82		82	36.91	0.21	22.89
15	37.82	0.50	5.10		49	43.26	0.46	26.04		83	36.37	0.02	10.67
16	33.44	0.10	13.63		50	34.94	0.13	23.86		84	10.92	0.09	27.54
17	30.02	0.43	10.38		51	17.90	0.01	14.71		85	11.34	0.11	26.62
18	17.89	0.20	-14.32		52	38.94	0.35	25.86		86	16.91	0.05	25.67
19	39.29	0.45	-13.01		53	10.15	0.37	10.68		87	41.93	0.27	23.84
20	17.77	0.26	-11.17		54	20.53	0.00	0.76		88	33.42	0.10	24.62
21	20.63	0.31	27.68		55	34.35	0.09	25.14		89	20.80	0.04	22.83
22	22.32	0.44	13.99		56	33.85	0.45	17.55		90	21.34	0.38	24.80
23	43.40	0.09	22.34		57	32.05	0.08	15.09		91	45.00	0.10	19.61
24	35.46	0.44	15.78		58	42.25	0.32	19.92		92	42.76	0.25	14.02
25	42.10	0.40	19.58		59	18.81	0.02	14.81		93	36.92	0.37	19.94
26	38.73	0.39	18.30		60	33.56	0.47	17.64		94	44.74	0.38	11.43
27	37.65	0.41	7.34		61	39.11	0.18	9.14		95	11.40	0.13	12.95
28	34.50	0.39	20.55		62	34.91	0.09	17.50		96	32.72	0.42	-13.82
29	41.23	0.43	-15.00		63	16.14	0.17	13.97		97	38.37	0.45	19.16
30	27.03	0.05	25.35		64	42.50	0.16	17.96		98	40.32	0.50	16.72
31	30.47	0.36	25.72		65	35.62	0.41	19.92		99	25.52	0.41	19.99
32	29.36	0.50	12.06		66	44.02	0.25	-15.00		100	27.50	0.40	27.27
33	11.64	0.36	15.31		67	28.95	0.34	21.34					
34	39.51	0.19	-15.00		68	32.46	0.45	11.24					

Table A5: Results of data matching, static data, Patient D

i = alveolar compartment number

i	TOP	k	P_{ext}		i	TOP	k			i	TOP	k	P_{ext}
1	38.86	0.28	20.87		35	45.00	0.30	28.50		69	5.00	0.15	1.48
2	10.66	0.38	-4.93		36	10.00	0.31	15.55		70	33.03	0.01	23.60
3	24.18	0.02	-13.43		37	27.40	0.08	8.87		71	39.31	0.11	19.90
4	21.19	0.10	-3.60		38	33.07	0.31	-15.00		72	14.33	0.36	20.54
5	5.00	0.36	-14.66		39	18.16	0.12	-11.25		73	34.31	0.35	9.38
6	25.61	0.45	-14.26		40	19.68	0.44	22.62		74	14.45	0.36	20.53
7	43.29	0.10	15.97		41	25.04	0.34	16.26		75	25.37	0.38	4.79
8	26.98	0.47	-12.88		42	44.80	0.06	21.90		76	25.02	0.42	23.23
9	35.94	0.49	-12.08		43	37.93	0.31	-9.09		77	40.98	0.20	14.47
10	45.00	0.49	-11.28		44	45.00	0.03	25.28		78	35.24	0.20	1.26
11	37.73	0.50	18.78		45	27.66	0.32	19.27		79	36.77	0.25	18.23
12	29.50	0.16	-1.63		46	40.13	0.27	-15.00		80	18.05	0.33	13.05
13	17.98	0.50	-15.00		47	22.07	0.26	15.33		81	29.69	0.05	10.24
14	41.00	0.02	-15.00		48	17.47	0.33	6.03		82	27.67	0.28	15.32
15	42.68	0.28	-14.27		49	26.30	0.06	27.24		83	21.24	0.02	22.83
16	5.00	0.50	7.30		50	33.35	0.29	15.87		84	12.85	0.22	21.16
17	25.24	0.14	-15.00		51	45.00	0.24	-10.42		85	16.79	0.35	21.23
18	23.93	0.12	19.74		52	34.58	0.31	-10.98		86	24.74	0.26	10.89
19	25.98	0.03	-12.18		53	29.86	0.02	22.86		87	29.66	0.00	19.79
20	42.80	0.21	-15.00		54	28.57	0.00	28.80		88	31.49	0.31	2.70
21	45.00	0.30	20.04		55	10.67	0.10	27.14		89	39.13	0.00	15.32
22	31.18	0.18	11.75		56	45.00	0.22	12.79		90	30.69	0.02	18.95
23	41.22	0.48	-4.09		57	12.53	0.11	8.25		91	16.74	0.04	6.43
24	20.61	0.09	-13.63		58	21.81	0.17	22.19		92	29.56	0.50	18.49
25	25.33	0.23	-10.82		59	42.97	0.24	14.04		93	25.34	0.00	15.72
26	37.80	0.18	-12.74		60	28.22	0.27	28.07		94	20.83	0.42	16.58
27	39.60	0.42	28.80		61	35.69	0.37	-15.00		95	42.12	0.50	19.17
28	39.30	0.17	-2.62		62	38.35	0.16	28.80		96	30.00	0.43	-9.53
29	25.33	0.42	-10.76		63	10.31	0.28	25.42		97	9.17	0.12	12.92
30	6.34	0.15	15.81		64	15.18	0.45	23.42		98	39.51	0.08	17.52
31	43.40	0.43	-2.38		65	25.13	0.00	-15.00		99	8.03	0.26	-10.97
32	20.19	0.22	20.79		66	27.87	0.00	22.40		100	21.21	0.26	28.80
33	22.64	0.32	-0.16		67	9.47	0.23	-2.88					
34	42.39	0.43	22.36		68	42.93	0.13	16.22					

Table A6: Results of data matching, static data, Patient E

i = alveolar compartment number

i	TOP	k	P_{ext}		i	TOP	k			i	TOP	k	P_{ext}
1	14.34	0.14	10.12		35	35.05	0.38	-8.15		69	14.25	0.09	4.68
2	25.95	0.01	-15.00		36	36.57	0.36	6.87		70	23.80	0.06	6.40
3	38.44	0.50	-11.23		37	39.99	0.03	9.20		71	34.55	0.31	-6.52
4	37.59	0.28	-15.00		38	25.77	0.36	-9.64		72	41.41	0.36	-5.44
5	7.56	0.38	-8.64		39	34.02	0.11	-0.84		73	26.28	0.31	6.99
6	39.77	0.21	-10.85		40	31.12	0.50	-9.53		74	41.23	0.22	-10.25
7	44.43	0.35	-9.32		41	27.77	0.02	9.78		75	35.33	0.02	-6.28
8	20.49	0.25	-15.00		42	41.76	0.14	4.07		76	32.05	0.00	6.90
9	31.45	0.28	-13.48		43	30.93	0.43	27.86		77	7.45	0.09	0.50
10	30.76	0.42	-13.16		44	36.90	0.14	-5.68		78	32.11	0.06	15.84
11	40.95	0.42	-11.56		45	41.99	0.34	6.90		79	36.08	0.29	-10.63
12	35.45	0.25	-15.00		46	45.00	0.08	20.40		80	34.12	0.27	-6.09
13	32.84	0.31	-4.83		47	37.52	0.34	1.98		81	44.82	0.22	-0.38
14	40.26	0.48	-14.16		48	36.14	0.05	0.12		82	5.19	0.08	-2.95
15	42.68	0.16	10.05		49	27.18	0.30	-13.64		83	26.50	0.35	-1.55
16	40.19	0.10	6.32		50	28.73	0.02	6.56		84	39.98	0.06	0.46
17	37.95	0.46	-6.87		51	34.72	0.29	-10.79		85	43.20	0.03	3.87
18	35.05	0.28	-12.57		52	34.06	0.05	12.27		86	19.36	0.00	2.76
19	25.27	0.45	-12.05		53	29.90	0.01	10.49		87	8.58	0.09	8.90
20	44.60	0.30	-15.00		54	43.66	0.00	8.52		88	22.96	0.25	-10.70
21	43.32	0.45	-0.11		55	36.22	0.12	16.27		89	26.33	0.34	-6.95
22	36.35	0.40	-11.54		56	25.00	0.42	9.39		90	28.91	0.00	13.82
23	35.95	0.27	-15.00		57	28.42	0.00	-0.44		91	26.17	0.30	-6.83
24	40.41	0.34	-14.91		58	42.46	0.42	-9.53		92	26.08	0.09	-1.60
25	32.93	0.42	-13.22		59	36.72	0.23	10.46		93	6.17	0.01	-5.02
26	27.24	0.37	-12.62		60	23.65	0.02	-2.71		94	29.13	0.30	8.94
27	45.00	0.34	-5.25		61	42.48	0.30	0.32		95	35.35	0.07	9.71
28	33.34	0.08	-12.47		62	42.42	0.00	6.90		96	41.99	0.00	7.07
29	6.73	0.30	-9.22		63	28.56	0.27	-1.57		97	32.77	0.07	7.77
30	40.19	0.49	-15.00		64	43.35	0.00	-4.21		98	16.39	0.05	8.43
31	39.38	0.00	-7.40		65	30.05	0.18	-4.40		99	37.40	0.30	-9.00
32	44.77	0.28	-11.73		66	42.91	0.31	-4.29		100	25.00	0.17	8.22
33	13.43	0.40	-6.92		67	33.06	0.12	-4.93					
34	9.09	0.04	-5.30		68	41.31	0.25	-10.38					
