# Supplementary material for: Evaluation of lung recruitment maneuvers in acute respiratory distress syndrome using computer simulation
Source: Crit Care. 2015 Jan 12;19(1):8. doi: 10.1186/s13054-014-0723-6 (PMC4329196; doi:10.1186/s13054-014-0723-6)
Supplement: Additional file 3: — Additional figures and experiments. The file reports the effects on PaO2/FIO2 of changes in hemoglobin levels, cardiac output, and FIO2 for the MRS-10 RM applied to patient A and presents some further model validation results. [file 13054_2014_723_MOESM3_ESM.pdf]

Evaluation of lung recruitment maneuvers in Acute Respiratory Distress Syndrome using computer simulation

Anup Das[1], Marc Chikhani[2], Oana Cole[2], Wenfei Wang[1], Tayyba Ali[2], Mainul Haque[2], Declan G. Bates[1] and Jonathan G. Hardman[2]

[1]School of Engineering, University of Warwick, CV4 7AL, UK

[2]School of Medicine, University of Nottingham, NG7 2UH, UK

Corresponding Author: Prof. Declan G. Bates, School of Engineering, University of Warwick, CV4 7AL, UK, Email: D.Bates@warwick.ac.uk

The online data supplement for this paper contains additional material that could not be included in the main text due to space limitations, and is divided into three files. The first file (additional file 1) describes in detail the simulation model employed in the paper. Additional file 2 presents the optimization strategy used in matching the model to the ARDS patient data and the parameter values for the different models. Additional file 3 reports the effects on PaO_2/F_IO_2 of changes in haemoglobin levels, cardiac output, and F_IO_2 for the MRS-10 RM applied to patient A and presents some further model validation results.

Additional File 3: Additional figures and experiments

The PF ratio observed in patient A with changes in the hemoglobin concentration, cardiac output and F_IO_2 for the MRS-10 RM is shown in Figure A3.

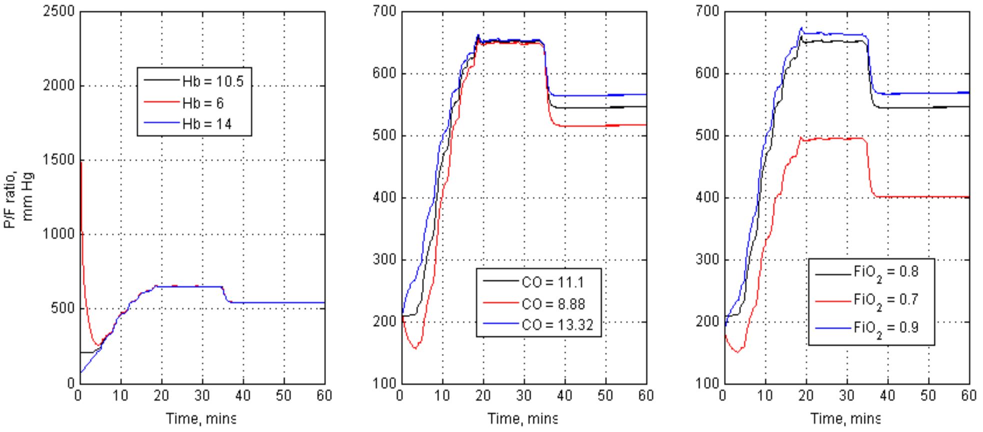

Figure A3: PaO_2/F_IO_2 resulting from changes in (left) haemoglobin levels (Hb, g dl^{-1}), (center) Cardiac output (CO, l min^{-1}) and (right) F_IO_2 for MRS-10 in patient A.

The dynamic compliance response of Patient A and Patient B for a PEEP titration procedure identical to the one given in Suarez-Sipmann 2007[1].

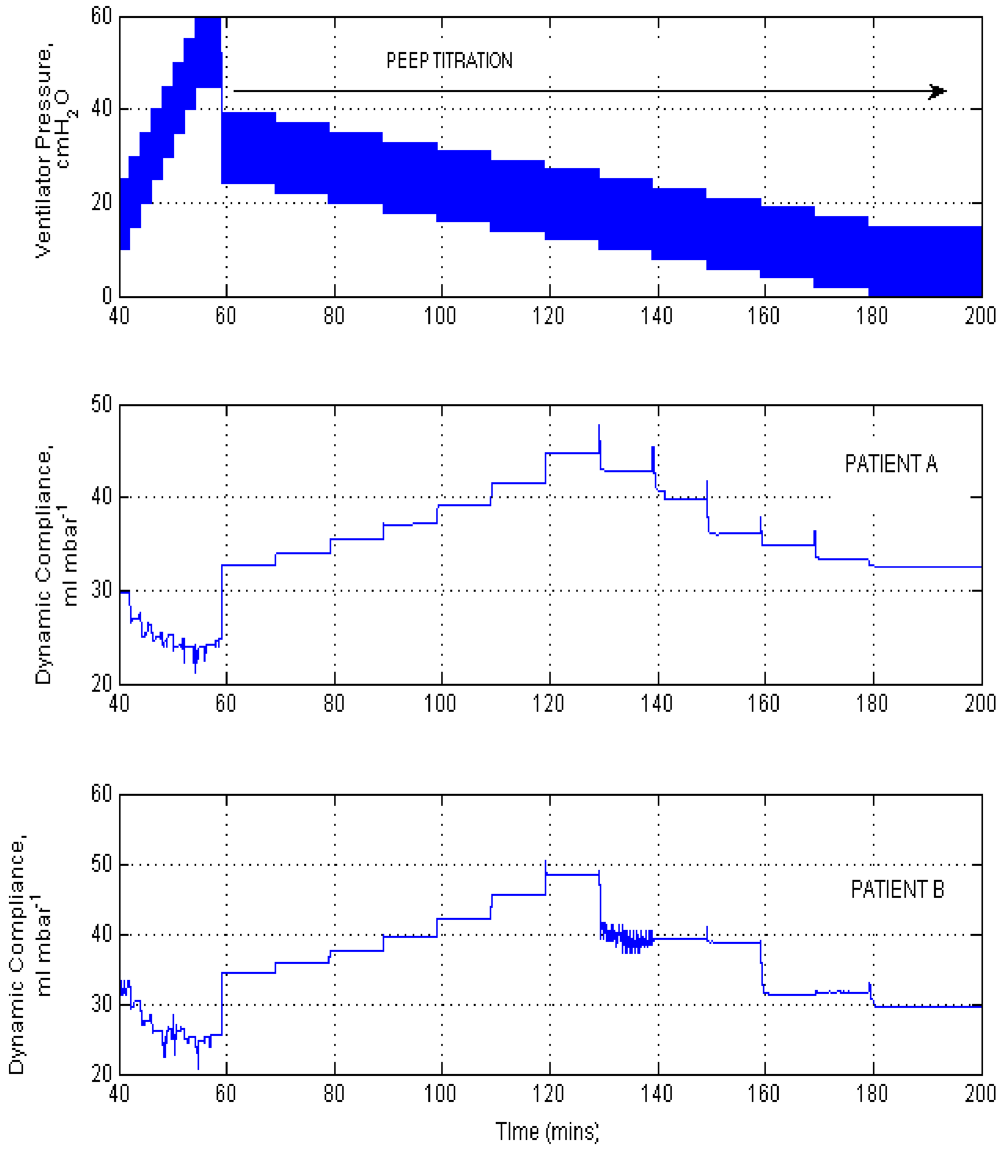

Figure A4: (top) The ventilator pressure waveform of PEEP titration after the recruitment phase, (center) the corresponding dynamic compliance response of the patient A and (bottom) the corresponding dynamic compliance of patient B.

---

[1] Suarez-Sipmann, Fernando, et al. Use of dynamic compliance for open lung positive end-expiratory pressure titration in an experimental study. *Critical care medicine* 2007;35(1): 214-221.
